# Predicting Anabolic Androgenic Steroid Doping among Specialized Health Care Patients with Elastic Net Regression Reveals Potential Laboratory Variables for “Patient Biological Passport”

**DOI:** 10.1186/s40798-025-00854-5

**Published:** 2025-05-01

**Authors:** Paula Katriina Vauhkonen, Jari Haukka, Ilkka Vauhkonen, Katarina Mercedes Lindroos, Mikko Ilari Mäyränpää

**Affiliations:** 1 Department of Forensic Medicine, University of Helsinki, Haartmaninkatu 3, P.O. Box 63, Helsinki, FI-00014 Finland; 2 Finnish Institute for Health and Welfare, Forensic Medicine unit, Mannerheimintie 166, P.O. Box 30, Helsinki, FI-00271 Finland; 3 Department of Public Health, University of Helsinki, Tukholmankatu 8 B, P.O. Box 20, Helsinki, FI-00014 Finland; 4 Novo Nordisk Farma Oy, Linnoitustie 6, Espoo, FI-02600 Finland; 5 Department of Pathology, University of Helsinki, Haartmaninkatu 3, P.O. Box 21, Helsinki, FI-00014 Finland; 6 Helsinki University Hospital, Diagnostic center, pathology, P.O. Box 340, Helsinki, FI-00029 Finland

**Keywords:** Doping, Anabolic androgenic steroids, AAS, Doping diagnostics, Doping biomarkers

## Abstract

**Background:**

Recent years have brought significant development in athlete doping use detection with the implementation of the Athlete Biological Passport (ABP). The aim of this study was to explore if similar methods could also be used to detect non-medical use of anabolic androgenic steroids (AAS) among clinical patients. For this purpose, six elastic net regression models were trained in a sample of Finnish specialized health care male patients (*N* = 2918; no doping = 1911, AAS doping = 1007), using different approaches to longitudinal laboratory measurements as predictive variables. The laboratory data was retrieved from the Hospital District of Helsinki and Uusimaa (HUS) data lake, and doping use status was defined by patient disclosure, recorded in digital medical record free texts. Length of observation time (e.g., time between the first and last laboratory measurement) was used as weight. Model performance was tested with holdout cross-validation.

**Results:**

All the tested models showed promising discriminative ability. The best fit was achieved by using the existence of out-of-reference range measurements of 31 laboratory parameters as predictors of AAS doping, with test data area under the receiver operating characteristic curve (AUC) of 0.757 (95% CI 0.725–0.789).

**Conclusions:**

The findings of this preliminary study suggest that AAS doping could be detected in clinical context using real-life longitudinal laboratory data. Further model development is encouraged, with added dimensions regarding the use of different AAS substances, length of doping use, and other background data that may further increase the diagnostic accuracy of these models.

**Supplementary Information:**

The online version contains supplementary material available at 10.1186/s40798-025-00854-5.

## Key Points

1. Clinical laboratory data can be used more efficiently in clinical doping use diagnostics.

2. Reference range deviations seem to be the most potential approach for further model development.

## Background

In the battle against doping in competitive sports, different approaches have been attempted in the past years to detect doping not only directly from biological samples but indirectly via hematological markers [1]. So far, individual longitudinal biomarker profiles seem to provide the most robust background for this task and have led to the development of the Athlete Biological Passport (ABP) [2, 3]. The ABP uses a Bayesian statistical approach to identify non-physiological variation in the athlete’s erythropoiesis-related hematological markers (the hematological module), androgen markers (the steroid module), and the recently incorporated endocrine markers (the endocrine module). In case of atypical marker variation, the results and background data (such as the athlete’s medical history, journeys, altitude training, etc.) are examined by human experts to decide if the passport is suspicious of doping or if other pathology might come into question [3–5].

In the field of medicine, there is also a need to better identify patients with doping– meaning the non-medical use of doping-classified substances for either competitive or recreational purposes. Previous research indicates that non-medical use of anabolic androgenic steroids (AAS)– the historically most popular doping substances among men in and out of competitive sports [6]– may lead to increased morbidity and mortality [7–9]. The medical issues related to AAS doping range from acute thrombotic emergencies to liver, kidney or heart failure and infertility, depending on the used substances and length of use [9–13]. Such medical conditions are routinely encountered in specialized health care, but the doctor may be unable to get a truthful anamnesis about the etiology behind the complaint, often due to fear of stigmatization [14–16]. Since direct laboratory testing for AAS is usually not clinically available, indirect detection of AAS doping via physical examination, laboratory markers and ultimately, patient disclosure, remain the only route to correct diagnosis.

To date, several biochemical markers indicative of AAS doping have been identified. The most unequivocal markers are the result of hypothalamic–pituitary–gonadal (HPG) axis suppression, observed as a decrease in serum luteinizing hormone (LH), follicle-stimulating hormone (FSH), and intratesticular and secreted testosterone (T) in males [12, 17]. After AAS use cessation, lowered serum T values may persist for several months [17, 18]. In addition, supra-physiological dosing of AAS often leads to noticeable increase in the erythropoiesis-related laboratory parameters (serum erythropoietin (EPO), hemoglobin (HGB), hematocrit (HCT) and reticulocyte percentage (RET%)) [9, 19]. Biochemical changes are also observed in lipoprotein markers, especially with orally administrable AAS (elevated low density cholesterol (LDL) and apolipoprotein B (ApoB); lowered high density cholesterol (HDL), apolipoprotein AI (ApoA1) and lipoprotein A (Lp(a))); liver and kidney markers (increased plasma alanine transaminase (ALT), alkaline phosphatase (ALP), gamma-glutamyl transpeptidase (GGT) and creatinine (Cr), lowered sex hormone binding globulin (SHBG)); and thrombotic markers (including changes in coagulation factors and higher D-dimer levels) [9, 13, 20–23]. There is also evidence of altered insulin sensitivity, that could clinically manifest as elevated blood glucose [24]. However, individual laboratory parameters of AAS using patients may be within the laboratory reference range, and those of non-AAS-using patients may fall out of the populational references due to various physiological and pathological causes [1, 25–28]. Therefore, more advanced biomarker-based diagnostic tools could be beneficial in detecting AAS doping in clinical context.

In this study we aimed to determine, if longitudinal biomarkers retrieved from a large clinical laboratory database could be utilized to predict AAS doping among specialized health care male patients, using elastic net regression [29]. This regression method combines the Lasso and Ridge penalties to optimize feature selection and reduction of overfitting, making it suitable for large datasets with several correlated variables. Like the ABP, such a predictive model could be used as a clinical support tool in detecting undisclosed AAS use in health care and help initiate discussion with the patient.

## Methods

The sample was gathered from the Hospital District of Helsinki and Uusimaa (HUS) data lake [30] using doping related key word search targeted to digital medical records. The data acquirement process is described in more detail in a previous publication [31]. In brief, the search covered all digital medical records dated between 2002 and 2020, including in- and outpatient specialized health care in HUS special catchment area of 2.2 million inhabitants. All medical records indicating discussions of doping use with the doctor were initially included. Personal identity code was used to identify birth date and sex, and to unite all available laboratory data from the data lake (2000–2021; sourced from several different clinical laboratories) to each patient. Utilizing short text sections retrieved from the medical records, male patients with acknowledged AAS doping (*N* = 1007) were separated manually by two researchers (PKV and KL) from those patients that had denied all doping (e.g., the use of any doping substances, *N* = 1911). Patients with inconclusive doping use status (e.g., suspicion of AAS doping without confronting the patient with the issue, records with misspelling, etc.; *N* = 98) were excluded by consensus.

The laboratory data included the test date and time, test result (numeral value for quantitative measurements and positive/negative for qualitative measurements), reference range provided by the analyzing laboratory, and whether the test result was outside (1) or within (0) the laboratory reference range. Results below the laboratory’s lower limit of quantification (LLOQ) were imputed using uniform distribution, and the maximum reported value was used for values above the laboratory’s upper limit of quantification (ULOQ). In case of several daily measurements/patient, a daily median was used. Finally, only laboratory tests with ≥ 50 individual patient measurements were included in further analysis, leaving 100 laboratory variables for model construction (SUPPLEMENT 1,* Laboratory variables.pdf*). In the AAS doping group, AAS use might have occurred at any point with respect to the observation period (e.g., time between the first and last available laboratory measurement in the dataset).

Statistical analyses were computed using R software (version 4.3.2) [32]. Prediction models for AAS doping were constructed with elastic net regression (R package “glmnet” [33, 34]). The outcome was binomial (no doping/AAS doping). We used the following approaches for prediction:

1. ***Indicator.****Indicator if a certain laboratory measurement was observed in the data set (no = 0*,* yes = 1).*
2. ***Frequency.****Frequency of laboratory measurements (0*,*1*,*2*,*…) in the data set.*
3. ***Variance***. *Variance of quantifiable laboratory measurements. Categorical variable with three levels*:
  • *Under Median*
  • *Over Median*
  • *missing (NA), e.g.*,* < 3 measurements/patient available.*
4. ***Variance excluding NA.****Variance of quantifiable laboratory measurements*,* with a penalty term to exclude missing (NA) values (e.g.*,* prediction not constructed on missing variance).*
5. ***Indicator out of reference range.****Indicator if a certain laboratory measurement was out of reference range in the data set (no = 0*,* yes = 1).*
6. ***Frequency out of reference range.****Frequency of out of reference range laboratory measurements (0*,*1*,*2*,*…) in the data set.*

Cross-validation was performed utilizing hold-out method, by dividing the dataset randomly into separate training (*N* = 1918 individuals) and testing (*N* = 1000 individuals) sets. Optimal combination of regularization parameter α and tuning parameter λ were determined using cross-validation within training set, and one standard error (SE) from the minimum λ value was used [35]. Length of observation interval (eg, time between the first and last laboratory measurement) was used as weight (in argument ”weights” in the glmnet function) to account for varying follow-up durations across patients, allowing those with a longer observation period to have greater influence on the fitted models. Predictions of elastic net models were studied using receiver operating characteristic (ROC) curves and by reporting area under the ROC curve (AUC) with 95% confidence intervals (CI) (R-package “pROC” [36]).

## Results

### Data Characteristics

Observation interval characteristics are presented in Table 1. Statistical summary of the laboratory variables (including individual laboratory marker distributions, number of available measurements per marker, and variance in the training data) is available in supplement 2 (SUPPLEMENT 2,* Laboratory summary.pdf*).

**Table 1:** Observation interval characteristics

	All (*N* = 2918)	No doping (*N* = 1911)	AAS doping (*N* = 1007)
Age at start of observation interval *years*,* mean (SD)*	32.0 (10.3)	31.6 (10.1)	32.6 (10.7)
Length of observation interval *years*, *mean (SD)*	7.0 (6.2)	6.1 (6.0)	8.7 (6.4)
Total number of laboratory measurements/patient, *Median (IQR)*	88.0 (282)	53.0 (186)	190.0 (420)

### Elastic Net Results

**Fig. 1:**
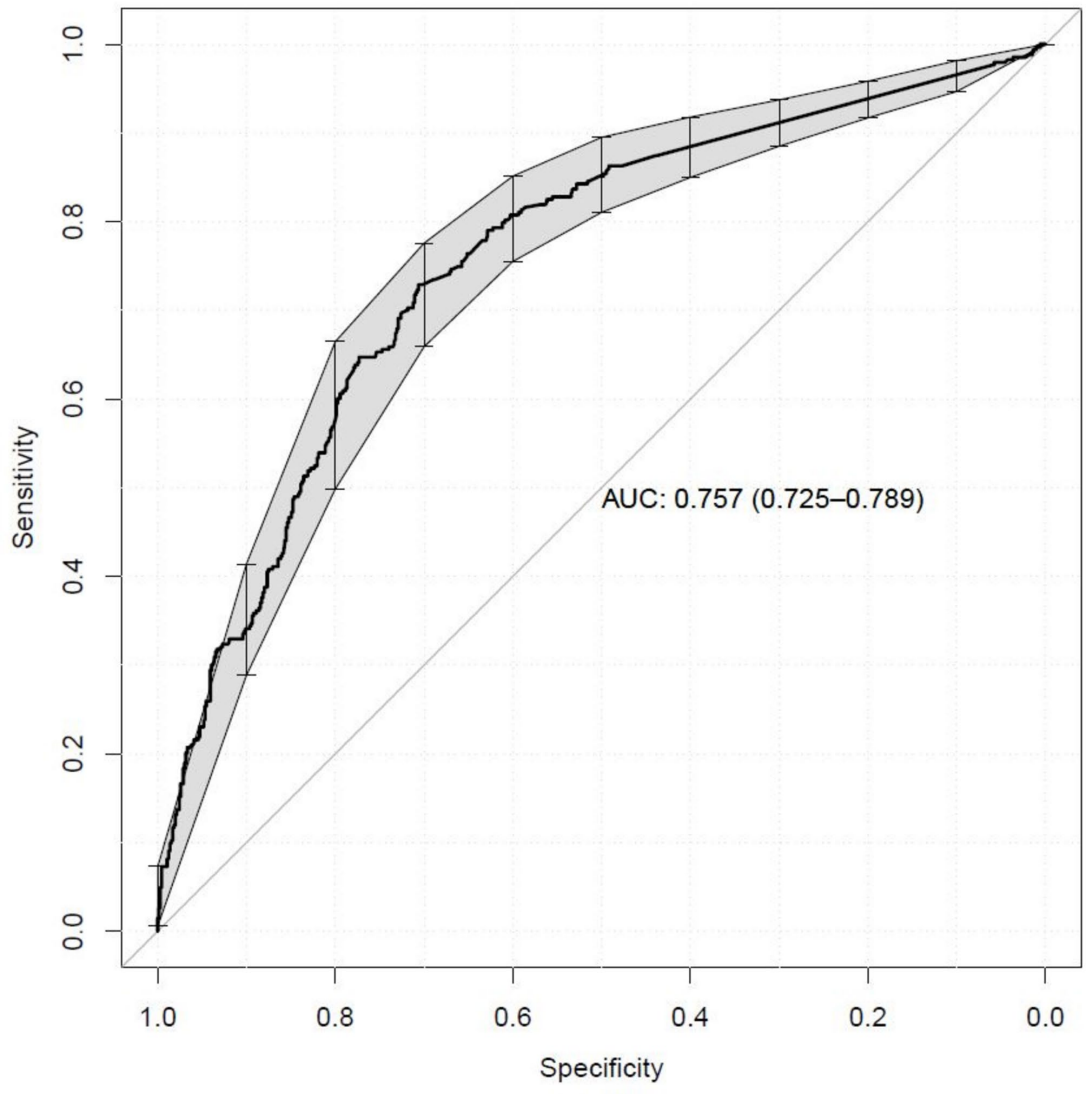
ROC curve, model no. 5, *Indicator out of reference range*. The model was built on an approach that predicted the patients’ AAS use status (AAS doping or no doping) using the existence of laboratory measurement reference range deviations as predictors. 95% confidence interval is marked with bars and shading

Model performances are presented in Table 2. All model predictors, odds ratios (OR) and ROC curves are available in supplement 3 (SUPPLEMENT 3,* Elastic net results.pdf*). The highest AUC was achieved with model no. 5, *Indicator out of reference range* (AUC 0.757 (95% CI 0.725–0.789); Table 3; Fig. 1). This model was run with four different random number seeds, yielding similar results with 19 variables common to all runs (data not shown).

**Table 2:** Model performances

Model no.	No. of variables	AUC (95% CI)
1. Indicator	24	0.740 (0.709–0.771)
2. Frequency	16	0.720 (0.688–0.752)
3. Variance	16	0.744 (0.711–0.776)
4. Variance excluding NA	10	0.734 (0.702–0.766)
5. Indicator out of reference range	31	0.757 (0.725–0.789)
6. Frequency out of reference range	14	0.718 (0.685–0.750)

**Table 3:** Variables, coefficients and odds ratios of model no. 5, *Indicator out of reference range*. Predictors increasing the odds for belonging to AAS doping group are **bolded**. The direction of reference range deviance in AAS doping group is categorized as “higher” or “lower” when only single direction is applicable; “majority higher” or “majority lower” when ≥ 95% of measurements in the sample were higher or lower than the laboratory reference range, respectively; otherwise “lower & higher”. For hepatitis A and C, reference range deviance = test positivity

Variable no.	Variable	Deviance direction	Coefficient	OR
1	**B -HGB**	lower & higher	0.341	1.406
2	**B -HCT**	lower & higher	0.235	1.265
3	**E -MCH**	lower & higher	0.045	1.046
4	**E -RDW**	higher	0.996	2.706
5	fP-Fe		-0.118	0.889
6	fP -TC		-0.024	0.977
7	**fP -HDL-C**	lower	0.181	1.198
8	fP-PTH		-0.646	0.524
9	fP -TG		-0.077	0.926
10	fS -Fol		-1.627	0.197
11	L -Baso(A)		-0.011	0.989
12	L -Mono(A)		-0.178	0.837
13	**P -Amyl**	higher	0.040	1.041
14	**P -CK**	majority higher	0.228	1.256
15	P -Ferrit		-0.060	0.942
16	P -FVIII.		-0.104	0.901
17	P -IgA		-0.983	0.374
18	P -IgM		-0.485	0.616
19	**P -Cr**	lower & higher	0.517	1.676
20	**P -Myogl**	higher	0.082	1.085
21	**P -Na**	lower & higher	0.007	1.007
22	**Pt-GFReEPI**	lower	0.050	1.051
23	**S -CDT**	higher	0.174	1.190
24	**Hepat-A**	positive	0.526	1.692
25	S -Cort		-0.905	0.404
26	**S -LH**	majority lower	0.246	1.279
27	S -Prot		-0.130	0.878
28	**S -T**	lower & higher	0.156	1.169
29	U -Acr		-0.236	0.790
30	**U -Cr**	lower & higher	0.416	1.515
31	**Hepat-C**	positive	0.526	1.693

Laboratory abbreviations: B -HGB = Blood hemoglobin concentration, B -HCT = Blood hematocrit, E -MCH = Erythrocyte mean corpuscular hemoglobin, E -RDW = Erythrocyte red cell distribution width, fP-Fe = Fasting plasma iron, fP -TC = Fasting plasma total cholesterol, fP -HDL-C = Fasting plasma high density cholesterol, fP -PTH = Fasting plasma parathyroid hormone, fP -TG = Fasting plasma triglycerides, fS -Fol = Fasting plasma folate, L -Baso(A) = Leukocyte blood differential test, basophil, L -Mono(A) = Leukocyte blood differential test, monocyte, P -Amyl = Plasma amylase, P -CK = Plasma creatine kinase, P -Ferrit = Plasma ferritin, P -FVIII. = Plasma factor VIII activity, P -IgA = Plasma immunoglobulin A, P -IgM = Plasma immunoglobulin M, P -Cr = Plasma creatinine, P -Myogl = Plasma myoglobin, P -Na = Plasma sodium, P -URA = Plasma urate, Pt-GFReEPI = Glomerular filtration rate using CKD-EPI equation, S -CDT = Serum carbohydrate-deficient transferrin, Hepat-A = Hepatitis A virus M-antibodies (S -HAVAbM) (if positive or > 0, then Hepatitis A test = positive), S -Cort = Serum cortisol, S -LH = Serum luteinizing hormone, S -Prot = Serum protein, S -T = Serum testosterone or serum testosterone by mass spectrometry, U -Acr = Urine albumin-to-creatinine ratio, U -Cr = Urine creatinine, Hepat-C = Serum Hepatitis C virus, antibodies (S -HCVAb); nucleic acid, quantitative (S -HCVNh); and nucleic acid, qualitative (S -HCVNhO) combined (if positive or > 0, then Hepatitis C test = positive)

To further clarify the approach, the *Indicator out of reference range* -model can be tested in a web application built with R package “shiny” (https://jari-haukka.shinyapps.io/DopingApp/) [37]. Population prevalence of 1% was used as default to calculate positive predictive value (PPV) and negative predictive value (NPV) for given predictions, but the application is provided with user options to adjust the population prevalence along with preferred sensitivity/specificity cut points for decision-making. For reference, demographic estimate of lifetime doping use prevalence among Finnish males was 1.5–1.8% during 2010–2018 [38–40]. For test use, optimum cutpoint was determined as minimum of: (1 − sensitivities)^2^+(1 − specificities)^2^ closest to the diagonal of ROC [41, 42].

## Discussion

In this study, longitudinal clinical laboratory data was used to predict AAS doping among specialized health care male patients with elastic net regression. Of the six explored approaches, the best predictive accuracy was achieved using the existence of out-of-reference range measurements as predictors of AAS doping. The discriminative ability of this model (AUC = 0.757) can be considered excellent [43], especially bearing in mind the context: rather than determining a specific disease state, the model prediction can be used to initiate– or re-initiate– conversation with the patient, and help in delineating relevant differential diagnostics in each case. This is clinically important, as early disclosure of AAS use may help in limiting unnecessary diagnostic tests, prevent applying unfruitful treatment protocols, and even reduce the risk of possible AAS– medicinal drug interactions. As this was an explorative study, the presented models must be considered preliminary, and further development is necessary to ensure utility in clinical context. However, the results were promising and demonstrate that clinical laboratory data can be used effectively for recognizing patient AAS doping.

The predictive variables within the out-of-reference range model seem congruent with previous scientific literature. In line with prior expectations, low HDL -C and aberration in S -LH and S -T increased the OR towards AAS doping. The S -T deviance may indeed manifest in both ways: even though AAS have a deteriorative effect on the HPG axis, the use of external testosterone may cause significant increase in measurable S -T. Also, the well-established effect of AAS on hematopoiesis most likely explains the higher deviance in red blood cell -related markers (B -HGB, B -HCT, E-MCH and E -RDW) among these patients. It should be noted however, that except for E -RDW, the deviance was not only due to values above the reference range. Interestingly, the odds for AAS doping were lowered with out-of-reference range measurements in fasting serum folate (fS -Fol) and iron status markers (fP-Fe, P -Ferrit), suggesting that red blood cell deviations are more likely explained by these deficiencies in the no-doping group.

In vitro, testosterone has been shown to dampen peripheral blood mononuclear cell immunoglobulin production [44] and a study published by Calabrese et al. in 1989 found an association with AAS -supplemented bodybuilding and a decrease in IgA -concentrations [45]. In our sample, aberrant plasma immunoglobulin A (P -IgA) and immunoglobulin M (P -IgM) markedly decreased the OR for AAS doping. As research on this topic is scarce, it seems reasonable to hypothesize that our finding reflects IgA and IgM pathology (e.g., nephropathy, liver disease) more common among the no-doping than AAS-doping patients in the sample. Besides these immunoglobins, other variables clearly decreasing the OR included out-of-reference range fasting plasma parathyroid hormone (fP -PTH) and serum cortisol (S -Cort), again indicating higher prevalence of organic endocrinological pathology among the non-doping patients compared to the AAS doping group.

A subset of variables increasing the odds of AAS doping in the out-of-reference range model may partly reflect abnormally high muscle mass, the use of creatine supplements, and/or intense exercise regimens practiced in this patient group. These variables include increased the plasma creatine kinase (P -CK), myoglobin (P -Myogl), creatinine (P -Cr) and lowered glomerular filtration rate (Pt-GFReEPI), all captured as individual predictors by the model. P -CK and P -Myogl are not only markers of pathological muscular damage or muscle tissue disease (e.g., rhabdomyolysis, trauma or myositis), but increased values may also result from strenuous exercise (such as extensive weight training). The P -CK activity is also proportional to muscle mass [46]. It has been postulated that exercise induced P -CK increase is even more pronounced with AAS use, possibly due to induced muscle cell membrane permeability or the athlete’s increased capability of overloading [47, 48]. However, not all studies have been able to replicate this finding [49, 50]. Evidence of direct toxic effect of AAS on muscle tissue is lacking, but case reports suggest that supraphysiological doses of AAS combined with strenuous exercise may induce localized injection-site as well as generalized rhabdomyolysis [51–54].

Some of the observed deviance in Pt-GFReEPI may reflect truly lowered glomerular filtration rate, especially considering the two-way deviance of urine creatinine (U -Cr) in the sample. In addition to kidney disease, other plausible interpretation of lowered U -Cr is urine dilution, due to excessive water intake or the use of doping masking agents. Similar factors might also play part in the predictive value of aberrant plasma sodium (P -Na) in the model. Subtle deviance in P -Na is a common occurrence in specialized health care due to various pathophysiological reasons, and with AAS using patients the aberrance may relate to strenuous exercise or water retention [55]. In rat studies, nandrolone decanoate– one of the most commonly used AAS–, has been shown to cause changes in serum sodium concentrations and increase water intake [56, 57]. Common to all of the aforementioned markers is susceptibility to changes in plasma volume, whether induced physiologically, pharmacologically or with the use of saline solutions [55]. The same holds for B -HGB, which is– by definition– a concentration measurement [58].

We also found markedly higher odds for AAS doping with hepatitis-C test positivity. This finding indicates increased risk for blood-born infections among these patients, that could be due to needle sharing in connection with intramuscular injection AAS use, or concurrent intravenous substance abuse [59, 60]. The odds were also higher with hepatitis-A test positivity, yet it should be noted that the S -HAVAbM test does not differentiate between infection and vaccine-induced immunity.

Moreover, a previous Finnish study of young males found an association between AAS use and weekly drunkenness as drinking style [61]. Increased alcohol intake could also explain the slightly higher OR with heightened plasma amylase (P -Amyl) and serum carbohydrate-deficient transferrin (S -CDT), the latter being a surrogate biomarker for excessive alcohol consumption [62]. Despite this marker’s high specificity for alcohol related pathology, genetic polymorphism of transferrin, congenital disorders of glycosylation and other severe liver disease may increase S -CDT levels [62]. However, no other hepatic markers emerged as predictors of AAS -doping in the out-of-reference range model, providing further support to the previous conception that the changes in liver enzymes induced by AAS are generally minute, and severe liver damage is rare [9, 11]. It should also be recognized that P -ALT and P -ALP are expressed in skeletal muscle tissue, and similarly to P -CK and P -Myogl, rise in these markers’ serum concentrations is physiological after intense exercise [26]. In these cases, P -GT remains within reference range [63].

The discriminative ability of the other tested approaches were also promising, but the clinical applicability is not as straight forward. This is especially true for models 1 *(Indicator*) and 2 (*Frequency*), as these most likely reflect laboratory tests commonly ordered and re-ordered in clinics with high AAS use in general (e.g., endocrinology and cardiology). Interpreting variance in models 3 (*Variance*) and 4 (*Variance excluding NA*) is also complicated. Compared to the ABP, non-biological variation (e.g., pre-analytical and analytical variation) is most likely higher in clinical samples, as the sampling and analytical procedures are not as robust [64]. Furthermore, the predictive value of non-existent (NA) variance simply means there is not enough data in one of the groups– not that there is no variance in these markers in the tested population. When the NA feature is diminished with a penalty, loss of information debases the model performance. This observation is to be expected in clinical context, where infrequent laboratory testing may not be able to accurately depict variance in biological markers, at least not as effectively as the ABP does. There is also lack of baseline data to correctly assess physiological intra-individual variation, highlighting the need to explore other approaches for indirect AAS use detection in health care. However, the variance models seemed capable of detecting fluctuation in the most commonly tested markers, possibly due to the AAS using patients being “on” and “off” AAS substances. These models also shared several common predictors with the out-of-reference range model, including E -RDW, P -CK and P -Cr (SUPPLEMENT 3,* Elastic net results.docx).*

## Limitations

Our study has several important limitations to recognize. First, patients that deny AAS use may actually be using these substances, so belonging to the no-doping group in the sample cannot be interpreted as ground truth of no AAS use, potentially biasing the results. The possible boundaries for AAS use confession in Finnish specialized health care have not yet been elucidated, but fear of punishment should not prevent patient disclosure, as there is no legislation prohibiting personal use of AAS substances. On the other hand, it is very unlikely that patients would falsely confess AAS use to their doctor, so belonging to AAS doping group in this study is unambiguous. Polypharmacy is another important issue, as people with doping use may take several other drugs along with AAS [59] and the use of insulin or growth hormone, for example, may induce changes in metabolic markers [65]. AAS use regimens may also vary between patients with regards to the used AAS substances and length of use, most likely modifying the impact on the tested markers. Furthermore, variability in laboratory testing frequency and pre-analytical factors in clinical settings hinders the ability to accurately assess physiological intra-individual variation in the tested markers, adding uncertainty to the presented models. It is noteworthy that in our sample, the AAS doping group had on average longer observation periods and higher number of obtained laboratory measurements than the no-doping group. The reason for this finding is not clear; it could be speculated that these patients need more thorough examination or regular laboratory check-ups more often than the no-doping patients. Such factors may affect the presented approaches, even though the differentiating likelihood of observing a certain laboratory measurement or a measurement deviance due to variable observation period lengths was considered in the tested models. Last, this was an explorative study of a single hospital district, and the sample was not stratified by clinical diagnoses, visited clinics or received medical treatment. This limits the generalizability of our findings to other healthcare systems and patient populations. In its current form, even the best model is thus not suitable for delineating differential diagnoses in clinical use. Future studies should consider expanding the dataset to include multiple healthcare systems and more detailed patient background information, which could improve the models’ discriminative ability and provide more nuanced insights into differential diagnostics.

## Conclusions

In this study, we predicted AAS doping among Finnish specialized health care male patients with elastic net regression, using a combination of longitudinal biomarkers retrieved from a clinical laboratory database. Our results indicate that longitudinal real-life clinical laboratory data can be efficiently utilized in identifying AAS doping among health care patients. The best diagnostic accuracy was achieved using out-of-reference range laboratory measurements as predictors of AAS use. After further development, this type of model could be implemented into a clinical laboratory information system as a diagnostic aid that alerts the physician about possible AAS doping. Previous research indicates that only one-third of AAS users seek help from physicians [66], which may predispose these patients to delayed diagnosis and poorer outcome. In our previous study, we found an increase in health care connections of AAS using patients prior to premature death [67], further highlighting the importance of health care systems’ capability to recognize and provide support for medical issues related to AAS use. Early AAS use identification, polite inquiry and consequent patient disclosure may have a direct impact on the given clinical care, and guide in tailoring of personalized intervention and follow-up for these patients [9]. Future research is encouraged to train similar machine algorithm models and pave the way for a “Patient Biological Passport”.

## Electronic Supplementary Material

Below is the link to the electronic supplementary material.

**Supplementary Material 1**: **Supplement 1**: Laboratory variables.pdf.

**Supplementary Material 2**: **Supplement 2**: Laboratory summary.pdf.

**Supplementary Material 3**: **Supplement 3**: Elastic net results.pdf.

## Data Availability

The data underlying this article will be shared on reasonable request to the corresponding author.

## Abbreviations

AAS: Anabolic androgenic steroids
AUC: Area under the receiver operating characteristic curve
ABP: Athlete biological passport
CI: Confidence interval
HUS: Hospital district of helsinki and uusimaa
HPG-axis: Hypothalamic–pituitary–gonadal axis
LLOQ: Laboratory’s lower limit of quantification
NA: Not available, missing value
NPV: Negative predictive value
OR: Odds ratio
PPV: Positive predictive value
ROC: Receiver operating characteristic
SE: Standard Error
ULOQ: Laboratory’s upper limit of quantification
