# Supplementary material for: Predicting Anabolic Androgenic Steroid Doping among Specialized Health Care Patients with Elastic Net Regression Reveals Potential Laboratory Variables for “Patient Biological Passport”
Source: Sports Med Open. 2025 May 1;11:46. doi: 10.1186/s40798-025-00854-5 (PMC12045897; doi:10.1186/s40798-025-00854-5)
Supplement: Supplementary file 1 — Supplementary Material 1: Supplement 1: Laboratory variables.pdf. [file 40798_2025_854_MOESM1_ESM.pdf]

Sports Medicine - Open

Predicting anabolic androgenic steroid doping among specialized health care patients with elastic net regression reveals potential laboratory variables for “Patient Biological Passport”

Paula Katriina Vauhkonen[1], Jari Haukka[2], Ilkka Vauhkonen[3], Katarina Mercedes Lindroos[4], Mikko Ilari Mäyränpää[5]

[1] Department of Forensic Medicine, University of Helsinki, P.O. Box 63 (Haartmaninkatu 3), FI-00014, Helsinki, Finland, and Finnish Institute for Health and Welfare, Forensic Medicine unit, P.O. Box 30 (Mannerheimintie 166), FI-00271, Helsinki, Finland.

[2] Finnish Institute for Health and Welfare, Forensic Medicine unit, P.O. Box 30 (Mannerheimintie 166), FI-00271, Helsinki, Finland and Department of Public Health, University of Helsinki, PL 20 (Tukholmankatu 8 B), 00014, Helsinki, Finland.

[3] Novo Nordisk Farma Oy, Linnoitustie 6, 02600 Espoo, Finland.

[4] Finnish Institute for Health and Welfare, Forensic Medicine unit, P.O. Box 30 (Mannerheimintie 166), FI-00271, Helsinki, Finland.

[5] Department of Pathology, University of Helsinki, P.O. Box 21 (Haartmaninkatu 3), FI-00014, Helsinki, Finland and Helsinki University Hospital, Diagnostic center, pathology, P.O. Box 340, FI-00029 Helsinki, Finland.

Laboratory variables

Finnish abbreviation	Prefix	English	English abbreviation	Notes
B -Baso	Blood	basophil count	B -Baso	
B -Eos	Blood	eosinophil count	B -Eos	
B -Erblast	Blood	erythroblast count	B -Erblast	
B -Eryt	Blood	erythrocyte/red blood cell count	B -RBC	
B -Hb	Blood	hemoglobin concentration	B -HGB	
B - HbA1c_comb	Blood	hemoglobin-A1c (combination variable)	B -HbA1c	B -HbA1c_comb = Blood glycosylated hemoglobin (B -GHbA1CM, B -HbA1c) and Blood glycosylated hemoglobin, finger prick test (B -HbA1cVT) results combined; using the formula B -HbA1c (mmol/mol) = B -GHbA1CM(%) x 10.93 - 23.50
B -HKR	Blood	hematocrit	B -HCT	
B -La	Blood	erythrocyte sedimentation rate	B -ESR	
B -Leuk	Blood	leukocyte/white blood cell count	B -WBC	
B -Ly	Blood	lymphocyte count	B -LYM	
B -Monos	Blood	monocyte count	B -MONO	
B -Neut	Blood	neutrophil count	B -NEUT	
B -Trom	Blood	platelet count	B -PLT	
E -MCH	Erythrocyte	mean corpuscular hemoglobin	E -MCH	
E -MCHC	Erythrocyte	mean corpuscular hemoglobin concentration	E -MCHC	
E -MCV	Erythrocyte	mean corpuscular volume	E -MCV	

Finnish abbreviation	Prefix	English	English abbreviation	Notes
E -RDW	Erythrocyte	red cell distribution width	E -RDW	
E -Retik	Erythrocyte	reticulocyte count	E -RET	
fP -Fe	Fasting plasma	iron	fP -Fe	
fP -Gluk	Fasting plasma	glucose	fP -Glu	
fP -Kol	Fasting plasma	total cholesterol	fP -TC	
fP -Kol-HDL	Fasting plasma	high density cholesterol	fP -HDL-C	
fP -Kol-LDL	Fasting plasma	low density cholesterol	fP -LDL-C	
fP -Laktaat	Fasting plasma	lactate	fP -LAC	
fP -NH4-ion	Fasting plasma	ammonium ion	fP -NH4-ion	
fP -PTH	Fasting plasma	parathyroid hormone	fP -PTH	
fP -Transf	Fasting plasma	transferrin	fP -TRF	
fP -Trfesat	Fasting plasma	transferrin saturation	fP -TS	
fP -Trigly	Fasting plasma	trigycerides	fP -TG	
fS -Folaat	Fasting serum	folate	fS -Fol	
Hepat -A		Hepatitis A virus	See notes	Serum Hepatitis A virus, M-antibodies (S -HAVAbM); if positive or > 0, then Hepatitis A test = positive
Hepat -B		Hepatitis B virus (combination variable)	See notes	Hepat-B = Serum Hepatitis B virus, c antigen antibodies (S -HBcAb); M-antibodies to c antigen (S -HBcAbM); s-antigen (S -HBsAg); nucleic acid, quantitative (S -HBVNh); and nucleic acid, qualitative (S -HBVNhO) combined (if positive or > 0, then Hepatitis B test = positive)

Finnish abbreviation	Prefix	English	English abbreviation	Notes
Hepat -C		Hepatitis C virus (combination variable)	See notes	Hepat-C = Serum Hepatitis C virus, antibodies (S -HCVAb); nucleic acid, quantitative (S -HCVNh); and nucleic acid, qualitative (S -HCVNhO) combined (if positive or > 0, then Hepatitis C test = positive)
L -Baso(A)	Leukocyte	blood differential test, basophil	L -Baso(A)	
L -Eos(A)	Leukocyte	blood differential test, eosinophil	L -Eos(A)	
L -Lymf(A)	Leukocyte	blood differential test, lymphocyte	L -Lymf(A)	
L -Mono(A)	Leukocyte	blood differential test, monocyte	L -Mono(A)	
L -Neut(A)	Leukocyte	blood differential test, neutrophil	L -Neut(A)	
P -AFOS	Plasma	alkaline phosphatase	P -ALP	
P -ALAT	Plasma	alanine transaminase	P -ALT	
P -Alb	Plasma	albumin	P -Alb	
P -Amyl	Plasma	amylase	P -Amyl	
P -AmylP	Plasma	pancreatic amylase	P -AmylP	
P -APTT	Plasma	activated partial thromboplastin clotting time	P -APTT	
P -ASAT	Plasma	aspartate transaminase	P -AST	
P -AT3	Plasma	antithrombin III	P -AT3	
P -Bil	Plasma	bilirubin	P -Bil	
P -Bil-Kj	Plasma	conjugated bilirubin	P -CB	
P -Ca	Plasma	calcium	P -Ca	

Finnish abbreviation	Prefix	English	English abbreviation	Notes
P -Ca-albk	Plasma	albumin corrected calcium; P -Ca-albk = P -Ca + 0,020 * (41,3 – (P -Alb))	P -Ca-albk	
P -CK	Plasma	creatine kinase	P -CK	
P -CK-MBm	Plasma	creatine phosphokinase-MB	P -CK-MBm	
P -Cl	Plasma	chloride	P -Cl	
P -CRP	Plasma	c-reactive protein	P -CRP	
P -Ferrit	Plasma	ferritin	P -Ferrit	
P -Fibr	Plasma	fibrinogen	P -Fibr	
P -FiDD	Plasma	D-dimer test	P -FiDD	
P -FVIII.	Plasma	factor VIII activity	P -FVIII.	
P -Gluk	Plasma	glucose	P -Glu	
P -GT	Plasma	glutamyl transferase	P -GT	
P -IgA	Plasma	immunoglobulin A	P -IgA	
P -IgG	Plasma	immunoglobulin G	P -IgG	
P -IgM	Plasma	immunoglobulin M	P -IgM	
P -K	Plasma	potassium	P -K	
P -Krea	Plasma	creatinine	P -Cr	
P -KysC	Plasma	cystatin C	P -cysC	
P -LD	Plasma	lactate dehydrogenase	P -LD	
P -Mg	Plasma	magnesium	P -Mg	
P -Myogl	Plasma	myoglobin	P -Myogl	
P -Na	Plasma	sodium	P -Na	
P -Pi	Plasma	phosphate	P -Pi	

Finnish abbreviation	Prefix	English	English abbreviation	Notes
P -PSA	Plasma	prostate-specific antigen	P -PSA	
P -PSA-V	Plasma	prostate-specific antigen, free fraction	P -PSA-V	
P -T4-V	Plasma	thyroxine, free fraction	P -FT4	
P -TfR	Plasma	transferrin receptor	P -TfR	
P -TnI	Plasma	troponin I	P -TnI	
P -Trombai	Plasma	thrombin time	P -TT	
P -TSH	Plasma	thyroid-stimulating hormone	P -TSH	
P -TT	Plasma	prothrombin time	P -PT	
P -Uraat	Plasma	urate	P -URA	
P -Urea	Plasma	urea	P -Urea	
Pt-GFReEPI		glomerular filtration rate using CKD-EPI equation	Pt-GFReEPI	
S -B12-Vit	Serum	vitamin B12	S -B12-Vit	
S -Ca-Ion_comb	Serum	ionized calcium (combination variable)	S -Ca-Ion_comb	S -Ca-Ion_comb = Plasma ionized calcium (P -Ca-Ion) and Serum ionized calcium (S -Ca-Ion) results combined
S -CDT	Serum	carbohydrate-deficient transferrin	S -CDT	
S -D-25	Serum	25-hydroxy vitamin D	S -D-25	
S -EPO	Serum	erythropoietin	S -EPO	

Finnish abbreviation	Prefix	English	English abbreviation	Notes
S -FSH	Serum	follicle stimulating hormone	S -FSH	
S -HIVAgAb	Serum	Human Immunodeficiency Virus antigen/antibodies	S -HIVAgAb	
S -Korsol	Serum	cortisol	S -Cort	
S -LH	Serum	luteinizing hormone	S -LH	
S -MPOAbG	Serum	myeloperoxidase, IgG antibodies	S -MPOAbG	
S -Prealb	Serum	prealbumin	S -Prealb	
S -PRL	Serum	prolactin	S -PRL	
S -Prot	Serum	protein	S -Prot	
S -SHBG	Serum	sex hormone binding globulin	S -SHBG	
S - Testo_comb	Serum	testosterone (combination variable)	S -T	S -Testo_comb = Serum testosterone (S -Testo) and Serum testosterone, by mass spectrometry (S -TestoMS) results combined
U -Alb	Urine	albumin	U -Alb	
U -AlbKre	Urine	albumin-to-creatinine ratio	U -Acr	
U -Krea	Urine	creatinine	U -Cr	
