# Supplementary material for: Predicting Anabolic Androgenic Steroid Doping among Specialized Health Care Patients with Elastic Net Regression Reveals Potential Laboratory Variables for “Patient Biological Passport”
Source: Sports Med Open. 2025 May 1;11:46. doi: 10.1186/s40798-025-00854-5 (PMC12045897; doi:10.1186/s40798-025-00854-5)
Supplement: Supplementary file 2 — Supplementary Material 2: Supplement 2: Laboratory summary.pdf. [file 40798_2025_854_MOESM2_ESM.pdf]

Sports Medicine - Open

Predicting anabolic androgenic steroid doping among specialized health care patients with elastic net regression reveals potential laboratory variables for “Patient Biological Passport”

Paula Katriina Vauhkonen[1], Jari Haukka[2], Ilkka Vauhkonen[3], Katarina Mercedes Lindroos[4], Mikko Ilari Mäyränpää[5]

[1] Department of Forensic Medicine, University of Helsinki, P.O. Box 63 (Haartmaninkatu 3), FI-00014, Helsinki, Finland, and Finnish Institute for Health and Welfare, Forensic Medicine unit, P.O. Box 30 (Mannerheimintie 166), FI-00271, Helsinki, Finland.

[2] Finnish Institute for Health and Welfare, Forensic Medicine unit, P.O. Box 30 (Mannerheimintie 166), FI-00271, Helsinki, Finland and Department of Public Health, University of Helsinki, PL 20 (Tukholmankatu 8 B), 00014, Helsinki, Finland.

[3] Novo Nordisk Farma Oy, Linnoitustie 6, 02600 Espoo, Finland.

[4] Finnish Institute for Health and Welfare, Forensic Medicine unit, P.O. Box 30 (Mannerheimintie 166), FI-00271, Helsinki, Finland.

[5] Department of Pathology, University of Helsinki, P.O. Box 21 (Haartmaninkatu 3), FI-00014, Helsinki, Finland and Helsinki University Hospital, Diagnostic center, pathology, P.O. Box 340, FI-00029 Helsinki, Finland.

Statistical summary of the laboratory markers

Laboratory marker distributions

Patients with no doping (n=1911), patients with AAS doping (n=1007)

For laboratory test abbreviations, please see SUPPLEMENT 1.

Doping status	Variable	Unit	Mean	SD	25% (1. quartile)	Median	75% (3. quartile)	Total number of measurements
No doping	B -Baso	E9/l	0.0373	0.0319	0.0200	0.0300	0.050	6580
AAS doping	B -Baso	E9/l	0.0372	0.0269	0.0200	0.0300	0.0500	5625
No doping	B -Eos	E9/l	0.1978	0.2061	0.0800	0.1500	0.250	7053
AAS doping	B -Eos	E9/l	0.2248	0.2559	0.0900	0.1700	0.2900	6013
No doping	B -Erblast	E9/l	0.0035	0.0565	0.0000	0.0000	0.000	6128
AAS doping	B -Erblast	E9/l	0.0039	0.0447	0.0000	0.0000	0.0000	5092
No doping	B -Eryt	E12/l	4.6053	0.8301	4.1300	4.7400	5.160	21660
AAS doping	B -Eryt	E12/l	4.6372	0.8659	4.0900	4.7500	5.2500	23464
No doping	B -Hb	g/l	137.8163	24.2028	124.0000	142.0000	154.000	22037

Doping status	Variable	Unit	Mean	SD	25% (1. quartile)	Median	75% (3. quartile)	Total number of measurements
AAS doping	B -Hb	g/l	138.0862	26.5435	119.0000	142.0000	157.0000	23955
No doping	B -HbA1c_comb	mmol/mol	46.1245	18.0400	34.0000	38.0000	57.000	1349
AAS doping	B -HbA1c_comb	mmol/mol	40.7110	15.9494	33.0000	36.0000	41.0000	991
No doping	B -HKR	%	40.1958	7.0470	36.0000	41.0000	45.000	25331
AAS doping	B -HKR	%	39.7987	7.8963	34.0000	41.0000	46.0000	29472
No doping	B -La	mm/h	10.6480	16.0875	2.0000	5.0000	10.000	3229
AAS doping	B -La	mm/h	14.0422	20.5568	2.0000	6.0000	15.0000	2511
No doping	B -Leuk	E9/l	7.6225	4.8377	5.2000	6.9000	9.000	21661
AAS doping	B -Leuk	E9/l	8.0920	4.6386	5.8000	7.4000	9.6000	23483
No doping	B -Ly	E9/l	1.8532	1.7608	1.2800	1.7700	2.300	6580
AAS doping	B -Ly	E9/l	1.9616	1.3150	1.3800	1.8500	2.3900	5625
No doping	B -Monos	E9/l	0.6236	0.2801	0.4400	0.5700	0.750	6580
AAS doping	B -Monos	E9/l	0.6621	0.2959	0.4600	0.6100	0.8100	5625
No doping	B -Neut	E9/l	4.2959	3.7416	2.4100	3.4350	5.110	9160
AAS doping	B -Neut	E9/l	4.4623	2.9194	2.6000	3.8100	5.5900	8452

Doping status	Variable	Unit	Mean	SD	25% (1. quartile)	Median	75% (3. quartile)	Total number of measurements
No doping	B -Trom	E9/l	244.3397	109.8720	188.0000	231.0000	283.000	21612
AAS doping	B -Trom	E9/l	243.0510	107.4734	181.0000	229.0000	288.0000	23337
No doping	E -MCH	pg	30.1567	2.2778	29.0000	30.0000	31.000	21636
AAS doping	E -MCH	pg	30.0109	3.1093	29.0000	30.0000	31.0000	23415
No doping	E -MCHC	g/l	337.4017	12.8961	330.0000	338.0000	346.000	21614
AAS doping	E -MCHC	g/l	336.2838	14.0359	328.0000	338.0000	346.0000	23402
No doping	E -MCV	fl	89.2892	6.0012	86.0000	89.0000	92.000	21644
AAS doping	E -MCV	fl	89.0571	6.1551	86.0000	89.0000	92.0000	23421
No doping	E -RDW	%	14.1148	2.0799	13.0000	14.0000	15.000	34048
AAS doping	E -RDW	%	14.6722	2.2036	13.0000	14.0000	15.0000	37164
No doping	E -Retik	%	1.9886	2.2091	1.0000	1.5000	2.100	818
AAS doping	E -Retik	%	1.7412	1.1915	1.1000	1.4000	2.1000	570
No doping	fP-Fe	umol/l	15.5634	9.6005	8.5500	14.0000	20.000	459
AAS doping	fP-Fe	umol/l	14.9949	10.8837	6.5000	12.4500	20.1500	332
No doping	fP-Gluk	mmol/l	6.0994	1.6904	5.3000	5.7000	6.300	1030

Doping status	Variable	Unit	Mean	SD	25% (1. quartile)	Median	75% (3. quartile)	Total number of measurements
AAS doping	fP-Gluk	mmol/l	5.8954	1.5710	5.2000	5.6000	6.1000	1208
No doping	fP-Kol	mmol/l	4.6881	1.2885	3.9000	4.6000	5.400	2057
AAS doping	fP-Kol	mmol/l	4.6078	1.3256	3.8000	4.5000	5.3000	2018
No doping	fP-Kol-HDL	mmol/l	1.2151	0.4049	0.9600	1.1700	1.430	1890
AAS doping	fP-Kol-HDL	mmol/l	1.0855	0.3945	0.8300	1.0500	1.2800	1910
No doping	fP-Kol-LDL	mmol/l	2.9305	1.1085	2.2000	2.8100	3.600	1881
AAS doping	fP-Kol-LDL	mmol/l	2.9950	1.0810	2.2925	2.9000	3.6000	1916
No doping	fP-Laktaat	mmol/l	1.3858	1.1861	0.8000	1.1000	1.600	4034
AAS doping	fP-Laktaat	mmol/l	1.3012	1.3151	0.7000	0.9000	1.4000	6908
No doping	fP-NH4-ion	umol/l	54.9124	33.8562	30.0000	49.0000	71.500	255
AAS doping	fP-NH4-ion	umol/l	66.9183	103.6861	29.7500	45.0000	61.5000	208
No doping	fP-PTH	ng/l	129.2540	155.1746	44.0000	72.0000	131.000	458
AAS doping	fP-PTH	ng/l	208.5686	260.7352	39.0000	91.0000	299.7500	216
No doping	fP-Transf	g/l	2.2838	0.5734	1.8500	2.2000	2.630	442
AAS doping	fP-Transf	g/l	2.4317	0.6551	1.9600	2.4250	2.8550	310

Doping status	Variable	Unit	Mean	SD	25% (1. quartile)	Median	75% (3. quartile)	Total number of measurements
No doping	fP-Trfesat	%	26.6310	15.8302	16.0000	24.0000	34.000	420
AAS doping	fP-Trfesat	%	24.8728	18.4728	11.0000	20.0000	34.0000	283
No doping	fP-Trigly	mmol/l	1.8264	1.6436	0.9300	1.3200	2.130	2120
AAS doping	fP-Trigly	mmol/l	1.8797	3.4962	0.9500	1.3400	2.0100	2162
No doping	fS-Folaat	nmol/l	15.1859	7.8835	8.9500	14.1000	19.300	73
AAS doping	fS-Folaat	nmol/l	17.4971	10.1973	9.4000	15.9000	21.0000	72
No doping	L -Baso(A)	%	0.6026	0.6040	0.0000	1.0000	1.000	6577
AAS doping	L -Baso(A)	%	0.5552	0.5905	0.0000	1.0000	1.0000	5625
No doping	L -Eos(A)	%	3.2051	2.6843	1.0000	3.0000	4.000	6577
AAS doping	L -Eos(A)	%	3.3717	3.0394	1.0000	3.0000	4.0000	5625
No doping	L -Lymf(A)	%	29.1361	11.9088	21.0000	29.0000	37.000	6577
AAS doping	L -Lymf(A)	%	29.4224	12.1451	21.0000	29.0000	38.0000	5625
No doping	L -Mono(A)	%	9.7298	3.7238	8.0000	9.0000	11.000	6577
AAS doping	L -Mono(A)	%	9.6048	3.6417	7.0000	9.0000	11.0000	5625
No doping	L -Neut(A)	%	57.1306	13.4038	48.0000	56.0000	65.000	6577

Doping status	Variable	Unit	Mean	SD	25% (1. quartile)	Median	75% (3. quartile)	Total number of measurements
AAS doping	L -Neut(A)	%	56.7847	14.0689	47.0000	56.0000	66.0000	5625
No doping	P -AFOS	U/l	99.8906	99.9998	60.0000	76.0000	104.000	8028
AAS doping	P -AFOS	U/l	92.5147	82.9426	56.0000	73.0000	99.0000	7109
No doping	P -ALAT	U/l	103.2135	409.1421	24.0000	38.0000	68.000	10873
AAS doping	P -ALAT	U/l	121.9036	607.1739	28.0000	46.0000	85.0000	10438
No doping	P -Alb	g/l	34.0365	7.8932	28.9000	35.1000	40.000	3114
AAS doping	P -Alb	g/l	33.3511	7.1942	28.8000	34.4000	39.0000	2538
No doping	P -Amyl	U/l	95.2784	169.4192	41.7500	57.0000	85.000	524
AAS doping	P -Amyl	U/l	146.7670	478.9157	37.0000	53.0000	84.0000	592
No doping	P -AmylP	U/l	49.6955	130.0964	18.0000	26.0000	36.000	1172
AAS doping	P -AmylP	U/l	44.8233	87.5053	18.0000	26.0000	39.0000	1445
No doping	P -APTT	s	32.9617	10.6732	27.0000	30.0000	35.000	840
AAS doping	P -APTT	s	40.5301	21.0979	27.0000	32.0000	45.0000	892
No doping	P -ASAT	U/l	106.5388	435.1493	26.0000	34.0000	57.500	4235
AAS doping	P -ASAT	U/l	107.0940	536.9683	28.0000	40.0000	71.0000	4743

Doping status	Variable	Unit	Mean	SD	25% (1. quartile)	Median	75% (3. quartile)	Total number of measurements
No doping	P -AT3	%	94.6205	25.7483	79.0000	100.0000	113.000	361
AAS doping	P -AT3	%	87.9589	29.3675	68.0000	88.0000	107.0000	299
No doping	P -Bil	umol/l	29.6590	69.7229	8.0000	12.0000	22.000	4487
AAS doping	P -Bil	umol/l	22.7163	50.2676	7.0782	11.1611	19.0000	4677
No doping	P -Bil-Kj	umol/l	25.8516	68.7789	2.0000	4.0000	11.000	1694
AAS doping	P -Bil-Kj	umol/l	17.1459	46.6995	2.4203	5.0000	9.0000	1545
No doping	P -Ca	mmol/l	2.3224	0.1352	2.2500	2.3300	2.410	782
AAS doping	P -Ca	mmol/l	2.2952	0.1523	2.2000	2.3100	2.4000	715
No doping	P -Ca-albk	mmol/l	2.4327	0.1294	2.3600	2.4300	2.510	374
AAS doping	P -Ca-albk	mmol/l	2.4051	0.1036	2.3300	2.4050	2.4800	272
No doping	P -CK	U/l	2212.4496	12834.6553	102.0000	195.0000	505.000	1017
AAS doping	P -CK	U/l	1307.6091	5959.1780	134.0000	306.0000	805.0000	1517
No doping	P -CK-MBm	ug/l	19.4410	46.0916	2.0000	4.0000	12.000	358
AAS doping	P -CK-MBm	ug/l	21.1644	66.5589	3.0000	5.0000	12.2500	536
No doping	P -Cl	mmol/l	106.2235	4.4675	103.0000	106.0000	109.000	3119

Doping status	Variable	Unit	Mean	SD	25% (1. quartile)	Median	75% (3. quartile)	Total number of measurements
AAS doping	P -Cl	mmol/l	107.3997	6.7938	104.0000	107.0000	110.0000	5481
No doping	P -CRP	mg/l	32.6945	57.4416	2.0035	6.0000	36.000	14850
AAS doping	P -CRP	mg/l	40.6843	64.0165	2.2793	9.0000	54.0000	16311
No doping	P -Ferrit	ug/l	395.5532	933.8580	80.0000	169.0000	356.250	564
AAS doping	P -Ferrit	ug/l	342.9090	962.8282	64.2500	149.0000	337.5000	334
No doping	P -Fibr	g/l	3.5664	1.7985	2.3000	3.1500	4.700	398
AAS doping	P -Fibr	g/l	4.2967	2.1808	2.6000	4.0000	5.6000	321
No doping	P -FiDD	mg/l	1.5881	6.2751	0.1244	0.3000	0.900	663
AAS doping	P -FiDD	mg/l	3.4852	8.4641	0.2343	0.8000	2.6000	783
No doping	P -FVIII.	%	179.2725	69.5960	133.2500	163.5000	217.000	422
AAS doping	P -FVIII.	%	203.8827	93.3062	138.5000	179.0000	249.5000	308
No doping	P -Gluk	mmol/l	6.9285	2.5241	5.5000	6.3000	7.500	7179
AAS doping	P -Gluk	mmol/l	7.1075	2.8188	5.6000	6.5000	7.8000	10162
No doping	P -GT	U/l	143.7833	209.2983	29.0000	63.0000	155.000	1809
AAS doping	P -GT	U/l	97.3964	197.7119	24.0000	42.0000	93.0000	2093

Doping status	Variable	Unit	Mean	SD	25% (1. quartile)	Median	75% (3. quartile)	Total number of measurements
No doping	P -IgA	g/l	2.1384	1.6243	1.1600	1.8600	2.890	333
AAS doping	P -IgA	g/l	2.0735	1.2892	1.3700	1.8700	2.3900	175
No doping	P -IgG	g/l	10.2310	4.2822	7.4000	9.7000	12.600	633
AAS doping	P -IgG	g/l	12.3430	6.4123	8.5250	11.1500	14.3000	258
No doping	P -IgM	g/l	1.1026	0.7188	0.5900	0.9400	1.500	319
AAS doping	P -IgM	g/l	0.9521	0.9757	0.4500	0.7800	1.1700	165
No doping	P -K	mmol/l	4.0438	0.4583	3.8000	4.0000	4.300	18803
AAS doping	P -K	mmol/l	4.0772	0.4790	3.8000	4.0000	4.3000	22715
No doping	P -Krea	umol/l	98.3029	80.6298	71.0000	82.0000	97.000	17077
AAS doping	P -Krea	umol/l	104.0328	123.3231	70.0000	84.0000	100.0000	17707
No doping	P -KysC	mg/l	1.4980	0.7451	0.9500	1.2400	1.960	205
AAS doping	P -KysC	mg/l	1.1629	0.3151	0.9300	1.1050	1.3475	102
No doping	P -LD	U/l	444.0325	918.8946	185.0000	222.0000	302.000	955
AAS doping	P -LD	U/l	340.2499	609.4781	179.0000	217.0000	280.0000	643
No doping	P -Mg	mmol/l	0.7912	0.1258	0.7100	0.7900	0.870	1235

Doping status	Variable	Unit	Mean	SD	25% (1. quartile)	Median	75% (3. quartile)	Total number of measurements
AAS doping	P -Mg	mmol/l	0.8025	0.1250	0.7300	0.8000	0.8700	1197
No doping	P -Myogl	ug/l	1857.8908	7026.4696	56.0000	165.0000	964.000	213
AAS doping	P -Myogl	ug/l	1204.8704	2838.4369	77.2500	203.5000	980.0000	454
No doping	P -Na	mmol/l	138.8259	3.4914	137.0000	139.0000	141.000	18319
AAS doping	P -Na	mmol/l	138.4470	4.4906	136.0000	139.0000	141.0000	22603
No doping	P -Pi	mmol/l	1.1054	0.3490	0.8825	1.0600	1.270	1834
AAS doping	P -Pi	mmol/l	1.1184	0.5153	0.8200	1.0000	1.2400	1829
No doping	P -PSA	ug/l	2.7671	14.7556	0.4600	0.8500	1.385	255
AAS doping	P -PSA	ug/l	5.3923	12.3800	0.5850	0.9800	4.8200	283
No doping	P -PSA-V	ug/l	0.5440	1.5355	0.2000	0.3200	0.500	105
AAS doping	P -PSA-V	ug/l	0.4377	0.4124	0.2000	0.3000	0.5000	137
No doping	P -T4-V	pmol/l	13.7972	3.3174	12.0000	13.0000	15.000	473
AAS doping	P -T4-V	pmol/l	13.4619	2.9372	12.0000	13.0000	15.0000	464
No doping	P -TfR	mg/l	4.0592	3.7313	1.9000	3.3000	4.600	193
AAS doping	P -TfR	mg/l	5.1549	7.0788	2.2000	3.4000	5.7000	155

Doping status	Variable	Unit	Mean	SD	25% (1. quartile)	Median	75% (3. quartile)	Total number of measurements
No doping	P -TnI	ng/l	983.3586	6971.5487	3.0000	11.0000	62.000	717
AAS doping	P -TnI	ng/l	921.5675	4415.9344	4.0000	13.0000	85.0000	761
No doping	P -Trombai	s	21.9699	10.2995	18.0000	20.0000	23.000	341
AAS doping	P -Trombai	s	27.3708	22.8975	18.0000	20.0000	23.0000	274
No doping	P -TSH	mU/l	2.1201	2.4119	1.0575	1.6650	2.430	672
AAS doping	P -TSH	mU/l	2.0351	1.6532	1.0800	1.6750	2.4900	736
No doping	P -TT	%	85.5636	27.6665	69.0000	87.0000	103.000	4240
AAS doping	P -TT	%	83.0833	27.4111	67.0000	84.0000	100.0000	4558
No doping	P -Uraat	umol/l	360.3398	128.3916	279.0000	356.0000	438.000	615
AAS doping	P -Uraat	umol/l	354.8933	144.7336	251.7500	334.0000	431.0000	356
No doping	P -Urea	mmol/l	10.0233	7.4367	4.9000	7.9000	12.300	1505
AAS doping	P -Urea	mmol/l	12.5604	10.3135	5.5500	8.4000	16.1000	1467
No doping	Pt-GFReEPI	ml/min/1.73 m^2	93.7891	27.8594	80.0000	100.0000	114.000	6168
AAS doping	Pt-GFReEPI	ml/min/1.73 m^2	90.4421	26.4918	76.0000	95.0000	110.0000	5797
No doping	S -B12-Vit	pmol/L	429.9398	201.0755	308.0000	390.0000	528.500	83

Doping status	Variable	Unit	Mean	SD	25% (1. quartile)	Median	75% (3. quartile)	Total number of measurements
AAS doping	S -B12-Vit	pmol/L	493.6324	229.1286	340.5000	440.0000	575.2500	139
No doping	S -Ca-Ion_comb	mmol/l/pH7.4	1.1980	0.0820	1.1700	1.2100	1.240	2603
AAS doping	S -Ca-Ion_comb	mmol/l/pH7.4	1.2001	0.1387	1.1600	1.2100	1.2500	2437
No doping	S -CDT	%	1.8736	0.7813	1.5000	1.7000	2.000	303
AAS doping	S -CDT	%	1.8231	0.6150	1.5000	1.7000	2.0000	515
No doping	S -D-25	nmol/l	68.0856	30.8372	45.0000	64.0000	86.000	362
AAS doping	S -D-25	nmol/l	74.0981	34.7150	47.0000	69.5000	91.0000	214
No doping	S -EPO	U/l	9.7895	14.5903	4.2000	6.8000	9.700	129
AAS doping	S -EPO	U/l	16.3147	24.6639	6.2250	10.7000	18.3500	82
No doping	S -FSH	IU/l	9.2348	12.9908	2.6000	5.0000	9.500	357
AAS doping	S -FSH	IU/l	4.5348	6.0949	0.4942	2.7000	5.6000	202
No doping	S -Korsol	nmol/l	339.8550	192.5965	212.5000	359.5000	466.250	432
AAS doping	S -Korsol	nmol/l	377.6827	198.3988	276.5000	385.0000	453.0000	195
No doping	S -LH	IU/l	5.8700	10.5460	2.1000	3.8500	5.900	382
AAS doping	S -LH	IU/l	2.5429	3.1974	0.1000	1.5500	4.1000	304

Doping status	Variable	Unit	Mean	SD	25% (1. quartile)	Median	75% (3. quartile)	Total number of measurements
No doping	S -MPOAbG	IU/ml	1.2651	1.4920	0.4874	0.7583	1.500	157
AAS doping	S -MPOAbG	IU/ml	1.4943	1.8500	0.4005	0.8470	1.9386	110
No doping	S -Prealb	mg/l	217.2249	95.4436	159.0000	229.0000	280.000	305
AAS doping	S -Prealb	mg/l	230.5352	96.0771	167.2500	232.5000	293.2500	220
No doping	S -PRL	mU/l	336.8748	254.9575	181.7500	253.5000	379.250	292
AAS doping	S -PRL	mU/l	377.6710	407.8292	160.0000	261.0000	435.5000	220
No doping	S -Prot	g/l	67.2722	7.7253	63.6000	68.8000	72.100	241
AAS doping	S -Prot	g/l	69.6774	9.0737	64.5250	68.8500	75.7750	234
No doping	S -SHBG	nmol/l	30.7269	32.7337	16.0000	24.0000	35.000	260
AAS doping	S -SHBG	nmol/l	27.6000	18.2322	15.2500	23.0000	35.0000	246
No doping	S -Testo_comb	nmol/l	14.3884	9.7868	8.2000	12.4000	18.900	812
AAS doping	S -Testo_comb	nmol/l	20.4522	39.0426	7.7000	12.5000	20.5500	723
No doping	U -Alb	mg/l	467.2747	1058.3155	4.5570	26.5000	385.750	464
AAS doping	U -Alb	mg/l	510.8053	1150.5899	8.0000	148.0000	587.2500	182
No doping	U -AlbKre	mg/mmol	82.5532	160.3415	0.8898	13.5000	93.650	339

Doping status	Variable	Unit	Mean	SD	25% (1. quartile)	Median	75% (3. quartile)	Total number of measurements
AAS doping	U -AlbKre	mg/mmol	58.4781	84.7227	1.9000	25.0000	74.4000	145
No doping	U -Krea	mmol/l	11.8281	8.4327	5.9000	9.4000	15.800	945
AAS doping	U -Krea	mmol/l	9.7332	7.0826	4.4000	8.2000	13.3000	3141

Number of available measurements/marker

Given as medians [IQR] in each group. For laboratory test abbreviations, please see SUPPLEMENT 1.

Variable	Total sample (n=2918)	No doping (n=1911)	AAS doping (n=1007)
B..Baso	0.00 [0.00, 2.00]	0.00 [0.00, 2.00]	1.00 [0.00, 3.00]
B..Eos	0.00 [0.00, 2.00]	0.00 [0.00, 2.00]	1.00 [0.00, 3.00]
B..Erblast	0.00 [0.00, 2.00]	0.00 [0.00, 1.00]	1.00 [0.00, 3.00]
B..Eryt	5.00 [1.00, 15.00]	3.00 [0.00, 10.00]	10.00 [4.00, 24.00]
B..Hb	5.00 [1.00, 16.00]	3.00 [0.00, 10.00]	10.00 [4.00, 25.00]
B..HbA1c_comb	0.00 [0.00, 0.00]	0.00 [0.00, 0.00]	0.00 [0.00, 1.00]
B..HKR	5.00 [1.00, 17.00]	3.00 [0.00, 11.00]	11.00 [4.00, 28.00]
B..La	0.00 [0.00, 1.00]	0.00 [0.00, 1.00]	1.00 [0.00, 2.00]
B..Leuk	5.00 [1.00, 15.75]	3.00 [0.00, 10.00]	10.00 [4.00, 24.00]
B..Ly	0.00 [0.00, 2.00]	0.00 [0.00, 2.00]	1.00 [0.00, 3.00]
B..Monos	0.00 [0.00, 2.00]	0.00 [0.00, 2.00]	1.00 [0.00, 3.00]
B..Neut	1.00 [0.00, 3.00]	0.00 [0.00, 2.00]	1.00 [0.00, 5.00]
B..Trom	5.00 [1.00, 15.00]	3.00 [0.00, 10.00]	10.00 [4.00, 24.00]
E..MCH	5.00 [1.00, 15.00]	3.00 [0.00, 10.00]	10.00 [4.00, 24.00]
E..MCHC	5.00 [1.00, 15.00]	3.00 [0.00, 10.00]	10.00 [4.00, 24.00]
E..MCV	5.00 [1.00, 15.00]	3.00 [0.00, 10.00]	10.00 [4.00, 24.00]
E..RDW	6.00 [0.00, 24.00]	4.00 [0.00, 16.00]	16.00 [4.00, 38.00]
E..Retik	0.00 [0.00, 0.00]	0.00 [0.00, 0.00]	0.00 [0.00, 0.00]
fP.Fe	0.00 [0.00, 0.00]	0.00 [0.00, 0.00]	0.00 [0.00, 0.00]
fP.Gluk	0.00 [0.00, 1.00]	0.00 [0.00, 0.00]	0.00 [0.00, 1.00]
fP.Kol	0.00 [0.00, 1.00]	0.00 [0.00, 1.00]	1.00 [0.00, 2.00]
fP.Kol.HDL	0.00 [0.00, 1.00]	0.00 [0.00, 1.00]	1.00 [0.00, 2.00]
fP.Kol.LDL	0.00 [0.00, 1.00]	0.00 [0.00, 1.00]	1.00 [0.00, 2.00]
fP.Laktaat	0.00 [0.00, 0.00]	0.00 [0.00, 0.00]	0.00 [0.00, 1.00]
fP.NH4.ion	0.00 [0.00, 0.00]	0.00 [0.00, 0.00]	0.00 [0.00, 0.00]
fP.PTH	0.00 [0.00, 0.00]	0.00 [0.00, 0.00]	0.00 [0.00, 0.00]
fP.Transf	0.00 [0.00, 0.00]	0.00 [0.00, 0.00]	0.00 [0.00, 0.00]
fP.Trfesat	0.00 [0.00, 0.00]	0.00 [0.00, 0.00]	0.00 [0.00, 0.00]

Variable	Total sample (n=2918)	No doping (n=1911)	AAS doping (n=1007)
fP.Trigly	0.00 [0.00, 1.00]	0.00 [0.00, 1.00]	1.00 [0.00, 2.00]
fS.Folaat	0.00 [0.00, 0.00]	0.00 [0.00, 0.00]	0.00 [0.00, 0.00]
L..Baso.A.	0.00 [0.00, 2.00]	0.00 [0.00, 2.00]	1.00 [0.00, 3.00]
L..Eos.A.	0.00 [0.00, 2.00]	0.00 [0.00, 2.00]	1.00 [0.00, 3.00]
L..Lymf.A.	0.00 [0.00, 2.00]	0.00 [0.00, 2.00]	1.00 [0.00, 3.00]
L..Mono.A.	0.00 [0.00, 2.00]	0.00 [0.00, 2.00]	1.00 [0.00, 3.00]
L..Neut.A.	0.00 [0.00, 2.00]	0.00 [0.00, 2.00]	1.00 [0.00, 3.00]
P..AFOS	1.00 [0.00, 4.00]	0.00 [0.00, 2.00]	2.00 [0.00, 6.00]
P..ALAT	1.00 [0.00, 6.00]	1.00 [0.00, 4.00]	4.00 [1.00, 10.00]
P..Alb	0.00 [0.00, 1.00]	0.00 [0.00, 0.00]	0.00 [0.00, 2.00]
P..Amyl	0.00 [0.00, 0.00]	0.00 [0.00, 0.00]	0.00 [0.00, 0.00]
P..AmylP	0.00 [0.00, 0.00]	0.00 [0.00, 0.00]	0.00 [0.00, 1.00]
P..APTT	0.00 [0.00, 0.00]	0.00 [0.00, 0.00]	0.00 [0.00, 1.00]
P..ASAT	0.00 [0.00, 2.00]	0.00 [0.00, 1.00]	1.00 [0.00, 5.00]
P..AT3	0.00 [0.00, 0.00]	0.00 [0.00, 0.00]	0.00 [0.00, 0.00]
P..Bil	0.00 [0.00, 2.00]	0.00 [0.00, 1.00]	1.00 [0.00, 3.00]
P..Bil.Kj	0.00 [0.00, 0.00]	0.00 [0.00, 0.00]	0.00 [0.00, 1.00]
P..Ca	0.00 [0.00, 0.00]	0.00 [0.00, 0.00]	0.00 [0.00, 0.00]
P..Ca.albk	0.00 [0.00, 0.00]	0.00 [0.00, 0.00]	0.00 [0.00, 0.00]
P..CK	0.00 [0.00, 1.00]	0.00 [0.00, 0.00]	0.00 [0.00, 2.00]
P..CK.MBm	0.00 [0.00, 0.00]	0.00 [0.00, 0.00]	0.00 [0.00, 0.00]
P..Cl	0.00 [0.00, 0.00]	0.00 [0.00, 0.00]	0.00 [0.00, 1.00]
P..CRP	3.00 [0.00, 10.00]	1.00 [0.00, 6.00]	6.00 [2.00, 16.00]
P..Ferrit	0.00 [0.00, 0.00]	0.00 [0.00, 0.00]	0.00 [0.00, 0.00]
P..Fibr	0.00 [0.00, 0.00]	0.00 [0.00, 0.00]	0.00 [0.00, 0.00]
P..FiDD	0.00 [0.00, 0.00]	0.00 [0.00, 0.00]	0.00 [0.00, 1.00]
P..FVIII.	0.00 [0.00, 0.00]	0.00 [0.00, 0.00]	0.00 [0.00, 0.00]
P..Gluk	0.00 [0.00, 3.00]	0.00 [0.00, 1.00]	2.00 [0.00, 6.00]
P..GT	0.00 [0.00, 1.00]	0.00 [0.00, 1.00]	1.00 [0.00, 2.00]
S..HIVAgAb	1.00 [0.00, 2.00]	1.00 [0.00, 2.00]	0.00 [0.00, 1.00]
P..IgA	0.00 [0.00, 0.00]	0.00 [0.00, 0.00]	0.00 [0.00, 0.00]
P..IgG	0.00 [0.00, 0.00]	0.00 [0.00, 0.00]	0.00 [0.00, 0.00]

Variable	Total sample (n=2918)	No doping (n=1911)	AAS doping (n=1007)
P..IgM	0.00 [0.00, 0.00]	0.00 [0.00, 0.00]	0.00 [0.00, 0.00]
P..K	3.00 [0.00, 11.00]	2.00 [0.00, 6.00]	7.00 [2.00, 18.00]
P..Krea	3.00 [1.00, 12.00]	2.00 [0.00, 8.00]	8.00 [3.00, 18.00]
P..KysC	0.00 [0.00, 0.00]	0.00 [0.00, 0.00]	0.00 [0.00, 0.00]
P..LD	0.00 [0.00, 0.00]	0.00 [0.00, 0.00]	0.00 [0.00, 0.00]
P..Mg	0.00 [0.00, 0.00]	0.00 [0.00, 0.00]	0.00 [0.00, 1.00]
P..Myogl	0.00 [0.00, 0.00]	0.00 [0.00, 0.00]	0.00 [0.00, 0.00]
P..Na	3.00 [0.00, 11.00]	2.00 [0.00, 6.00]	7.00 [2.00, 18.00]
P..Pi	0.00 [0.00, 0.00]	0.00 [0.00, 0.00]	0.00 [0.00, 0.00]
P..PSA	0.00 [0.00, 0.00]	0.00 [0.00, 0.00]	0.00 [0.00, 0.00]
P..PSA.V	0.00 [0.00, 0.00]	0.00 [0.00, 0.00]	0.00 [0.00, 0.00]
P..T4.V	0.00 [0.00, 0.00]	0.00 [0.00, 0.00]	0.00 [0.00, 0.00]
P..TfR	0.00 [0.00, 0.00]	0.00 [0.00, 0.00]	0.00 [0.00, 0.00]
P..TnI	0.00 [0.00, 0.00]	0.00 [0.00, 0.00]	0.00 [0.00, 0.00]
P..Trombai	0.00 [0.00, 0.00]	0.00 [0.00, 0.00]	0.00 [0.00, 0.00]
P..TSH	0.00 [0.00, 0.00]	0.00 [0.00, 0.00]	0.00 [0.00, 1.00]
P..TT	0.00 [0.00, 2.00]	0.00 [0.00, 1.00]	1.00 [0.00, 4.00]
P..Uraat	0.00 [0.00, 0.00]	0.00 [0.00, 0.00]	0.00 [0.00, 0.00]
P..Urea	0.00 [0.00, 0.00]	0.00 [0.00, 0.00]	0.00 [0.00, 0.00]
Pt.GFReEPI	0.00 [0.00, 4.00]	0.00 [0.00, 2.00]	1.00 [0.00, 6.00]
S..B12.Vit	0.00 [0.00, 0.00]	0.00 [0.00, 0.00]	0.00 [0.00, 0.00]
S..Ca.Ion_comb	0.00 [0.00, 1.00]	0.00 [0.00, 1.00]	0.00 [0.00, 1.50]
S..CDT	0.00 [0.00, 0.00]	0.00 [0.00, 0.00]	0.00 [0.00, 0.00]
S..D.25	0.00 [0.00, 0.00]	0.00 [0.00, 0.00]	0.00 [0.00, 0.00]
S..EPO	0.00 [0.00, 0.00]	0.00 [0.00, 0.00]	0.00 [0.00, 0.00]
S..FSH	0.00 [0.00, 0.00]	0.00 [0.00, 0.00]	0.00 [0.00, 0.00]
S..HAVAbM	0.00 [0.00, 0.00]	0.00 [0.00, 0.00]	0.00 [0.00, 0.00]
S..Korsol	0.00 [0.00, 0.00]	0.00 [0.00, 0.00]	0.00 [0.00, 0.00]
S..LH	0.00 [0.00, 0.00]	0.00 [0.00, 0.00]	0.00 [0.00, 0.00]
S..MPOAbG	0.00 [0.00, 0.00]	0.00 [0.00, 0.00]	0.00 [0.00, 0.00]
S..Prealb	0.00 [0.00, 0.00]	0.00 [0.00, 0.00]	0.00 [0.00, 0.00]
S..PRL	0.00 [0.00, 0.00]	0.00 [0.00, 0.00]	0.00 [0.00, 0.00]

Variable	Total sample (n=2918)	No doping (n=1911)	AAS doping (n=1007)
S..Prot	0.00 [0.00, 0.00]	0.00 [0.00, 0.00]	0.00 [0.00, 0.00]
S..SHBG	0.00 [0.00, 0.00]	0.00 [0.00, 0.00]	0.00 [0.00, 0.00]
S..Testo_comb	0.00 [0.00, 0.00]	0.00 [0.00, 0.00]	0.00 [0.00, 1.00]
U..Alb	0.00 [0.00, 0.00]	0.00 [0.00, 0.00]	0.00 [0.00, 0.00]
U..AlbKre	0.00 [0.00, 0.00]	0.00 [0.00, 0.00]	0.00 [0.00, 0.00]
U..Krea	0.00 [0.00, 0.00]	0.00 [0.00, 0.00]	0.00 [0.00, 1.00]
Hepat.B	1.00 [0.00, 2.00]	2.00 [0.00, 3.00]	0.00 [0.00, 2.00]
Hepat.C	1.00 [0.00, 2.00]	1.00 [0.00, 2.00]	0.00 [0.00, 1.00]

Variance in the training data (n=1918)

The table represents group differences with respect to the median variance in the whole training sample.

For example, in the marker "B..Baso", median variance in the sample is 0.000157. In the "No doping" group, 113 individuals (9.0%) has intra-individual variance greater than this, while in the "AAS doping" group, 94 individuals (14.2%) has greater variance. Thus, individuals in the "AAS doping group" tend to have greater variance in blood basophils, compared to individuals in the "No doping" group.

For laboratory test abbreviations, please see SUPPLEMENT 1.

Variable	Variance (median)	Number (%) of cases with intra-individual variance over median	
		No doping (n=1254)	AAS doping (n=664)
B..Baso	0.000157	113 (9.0)	94 (14.2)
B..Eos	0.00746	113 (9.0)	104 (15.7)
B..Erblast	0.0	23 (1.8)	27 (4.1)
B..Eryt	0.112	255 (20.3)	328 (49.4)
B..Hb	101	265 (21.1)	321 (48.3)
B..HbA1c_comb	7.33	40 (3.2)	33 (5.0)
B..HKR	8.41	264 (21.1)	324 (48.8)
B..La	17.7	75 (6.0)	68 (10.2)
B..Leuk	4.21	277 (22.1)	306 (46.1)
B..Ly	0.184	109 (8.7)	98 (14.8)
B..Monos	0.0264	104 (8.3)	103 (15.5)
B..Neut	2.73	123 (9.8)	139 (20.9)
B..Trom	1.24e.03	265 (21.1)	317 (47.7)
E..MCH	0.479	273 (21.8)	310 (46.7)
E..MCHC	52.2	278 (22.2)	305 (45.9)
E..MCV	3.28	271 (21.6)	312 (47.0)
E..RDW	0.27	252 (20.1)	342 (51.5)
E..Retik	0.0833	12 (1.0)	11 (1.7)
fP.Fe	28.4	10 (0.8)	7 (1.1)
fP.Gluk	0.173	37 (3.0)	51 (7.7)
fP.Kol	0.394	70 (5.6)	76 (11.4)

Variable	Variance (median)	Number (%) of cases with intra-individual variance over median	
		No doping (n=1254)	AAS doping (n=664)
fP.Kol.HDL	0.0296	59 (4.7)	77 (11.6)
fP.Kol.LDL	0.3	65 (5.2)	72 (10.8)
fP.Laktaat	0.156	52 (4.1)	64 (9.6)
fP.NH4.ion	0.235	6 (0.5)	8 (1.2)
fP.PTH	0.814	10 (0.8)	4 (0.6)
fP.Transf	0.09	9 (0.7)	7 (1.1)
fP.Trfesat	77.8	11 (0.9)	5 (0.8)
fP.Trigly	0.18	72 (5.7)	83 (12.5)
L..Baso.A.	0.23	111 (8.9)	96 (14.5)
L..Eos.A.	2.17	104 (8.3)	103 (15.5)
L..Lymf.A.	56.5	105 (8.4)	102 (15.4)
L..Mono.A.	3.66	107 (8.5)	100 (15.1)
L..Neut.A.	80.1	105 (8.4)	102 (15.4)
P..AFOS	202	136 (10.8)	150 (22.6)
P..ALAT	455	171 (13.6)	221 (33.3)
P..Alb	14.5	50 (4.0)	75 (11.3)
P..Amyl	343	12 (1.0)	18 (2.7)
P..AmylP	55.5	29 (2.3)	34 (5.1)
P..APTT	7.93	26 (2.1)	18 (2.7)
P..ASAT	310	84 (6.7)	142 (21.4)
P..AT3	154	8 (0.6)	11 (1.7)
P..Bil	22.5	89 (7.1)	101 (15.2)
P..Bil.Kj	4.78	42 (3.3)	39 (5.9)
P..Ca	0.00812	14 (1.1)	16 (2.4)
P..Ca.albk	0.0072	5 (0.4)	4 (0.6)
P..CK	6.15e.04	24 (1.9)	64 (9.6)
P..CK.MBm	36.8	11 (0.9)	24 (3.6)
P..Cl	5.24	37 (3.0)	61 (9.2)
P..CRP	423	238 (19.0)	253 (38.1)
P..Ferrit	5.91e.03	10 (0.8)	12 (1.8)
P..Fibr	1.34	11 (0.9)	8 (1.2)

Variable	Variance (median)	Number (%) of cases with intra-individual variance over median	
		No doping (n=1254)	AAS doping (n=664)
P..FiDD	0.37	18 (1.4)	25 (3.8)
P..FVIII.	2.22e.03	9 (0.7)	11 (1.7)
P..Gluk	1.07	103 (8.2)	140 (21.1)
P..GT	414	61 (4.9)	69 (10.4)
P..IgA	0.157	11 (0.9)	3 (0.5)
P..IgG	2.46	9 (0.7)	7 (1.1)
P..IgM	0.0317	8 (0.6)	5 (0.8)
P..K	0.0899	218 (17.4)	275 (41.4)
P..Krea	83.4	204 (16.3)	317 (47.7)
P..KysC	0.0436	6 (0.5)	3 (0.5)
P..LD.	1.42e.03	14 (1.1)	12 (1.8)
P..Mg	0.00733	23 (1.8)	34 (5.1)
P..Myogl	1.84e.05	8 (0.6)	18 (2.7)
P..Na	4.92	228 (18.2)	263 (39.6)
P..Pi	0.0372	33 (2.6)	41 (6.2)
P..PSA	1.85	7 (0.6)	7 (1.1)
P..PSA.V	0.00603	3 (0.2)	3 (0.5)
P..T4.V	3.12	12 (1.0)	11 (1.7)
P..TfR	2.82	6 (0.5)	6 (0.9)
P..TnI	286	26 (2.1)	28 (4.2)
P..Trombai	3.5	6 (0.5)	7 (1.1)
P..TSH	0.285	22 (1.8)	21 (3.2)
P..TT	224	104 (8.3)	124 (18.7)
P..Uraat	4.6e.03	13 (1.0)	8 (1.2)
P..Urea	5.92	29 (2.3)	31 (4.7)
Pt.GFReEPI	64.6	117 (9.3)	155 (23.3)
S..Ca.Ion_comb	0.00123	46 (3.7)	64 (9.6)
S..CDT	0.07	10 (0.8)	16 (2.4)
S..D	25.281	13 (1.0)	9 (1.4)
S..EPO	6.81	2 (0.2)	1 (0.2)
S..FSH	3.66	6 (0.5)	5 (0.8)

Variable	Variance (median)	Number (%) of cases with intra-individual variance over median	
		No doping (n=1254)	AAS doping (n=664)
S..Korsol	2.27e.04	14 (1.1)	2 (0.3)
S..LH	3.89	9 (0.7)	9 (1.4)
S..MPOAbG	0.686	1 (0.1)	3 (0.5)
S..Prealb	1.27e.03	11 (0.9)	8 (1.2)
S..PRL	3.96e.04	7 (0.6)	5 (0.8)
S..Prot	17.3	5 (0.4)	3 (0.5)
S..SHBG	23.6	6 (0.5)	6 (0.9)
S..Testo_comb	43.5	19 (1.5)	32 (4.8)
U..Alb	6.46e.03	15 (1.2)	7 (1.1)
U..AlbKre	1.5e.03	11 (0.9)	4 (0.6)
U..Krea	23.8	14 (1.1)	48 (7.2)
