# Supplementary material for: Predicting Anabolic Androgenic Steroid Doping among Specialized Health Care Patients with Elastic Net Regression Reveals Potential Laboratory Variables for “Patient Biological Passport”
Source: Sports Med Open. 2025 May 1;11:46. doi: 10.1186/s40798-025-00854-5 (PMC12045897; doi:10.1186/s40798-025-00854-5)
Supplement: Supplementary file 3 — Supplementary Material 3: Supplement 3: Elastic net results.pdf. [file 40798_2025_854_MOESM3_ESM.pdf]

Sports Medicine - Open

Predicting anabolic androgenic steroid doping among specialized health care patients with elastic net regression reveals potential laboratory variables for “Patient Biological Passport”

Paula Katriina Vauhkonen[1], Jari Haukka[2], Ilkka Vauhkonen[3], Katarina Mercedes Lindroos[4], Mikko Ilari Mäyränpää[5]

[1] Department of Forensic Medicine, University of Helsinki, P.O. Box 63 (Haartmaninkatu 3), FI-00014, Helsinki, Finland, and Finnish Institute for Health and Welfare, Forensic Medicine unit, P.O. Box 30 (Mannerheimintie 166), FI-00271, Helsinki, Finland.

[2] Finnish Institute for Health and Welfare, Forensic Medicine unit, P.O. Box 30 (Mannerheimintie 166), FI-00271, Helsinki, Finland and Department of Public Health, University of Helsinki, PL 20 (Tukholmankatu 8 B), 00014, Helsinki, Finland.

[3] Novo Nordisk Farma Oy, Linnoitustie 6, 02600 Espoo, Finland.

[4] Finnish Institute for Health and Welfare, Forensic Medicine unit, P.O. Box 30 (Mannerheimintie 166), FI-00271, Helsinki, Finland.

[5] Department of Pathology, University of Helsinki, P.O. Box 21 (Haartmaninkatu 3), FI-00014, Helsinki, Finland and Helsinki University Hospital, Diagnostic center, pathology, P.O. Box 340, FI-00029 Helsinki, Finland.

Elastic net results

Common English abbreviations (Engl. Abbr.) are given, when applicable. Comprehensive list of included laboratory measurements and abbreviations is available in supplement 1, *Laboratory variables.docx.*

1. Indicator

Variable no.	Variable	Engl. Abbr.	Coefficient	OR
1	E -RDW		0.1443864	1.1553304
2	E -Retik	E -RET	-0.2096019	0.8109070
3	fP-Laktaat	fP -LAC	0.1595842	1.1730231
4	fP-PTH		-0.3064936	0.7360232
5	P -AFOS	P -ALP	0.0220399	1.0222846
6	P -AmylP		0.0997601	1.1049058
7	P -ASAT	P -AST	0.1226524	1.1304914
8	P -Bil		0.1006147	1.1058505
9	P -CK		0.3138552	1.3686916
10	P -Cl		0.0479779	1.0491475
11	P -FiDD		0.4033148	1.4967780
12	P -GT		0.0077255	1.0077554
13	S -HIVAgAb		-0.0151537	0.9849606
14	P -IgA		-0.1355420	0.8732425
15	P -Krea	P -Cr	0.0229326	1.0231976
16	P -Na		0.0682179	1.0705986
17	P -TT	P -PT	0.1254030	1.1336052
18	S -CDT		0.3825191	1.4659729
19	Hepat-A		0.2927465	1.3401031
20	S -Korsol	S -Cort	-0.1884308	0.8282579
21	S -SHBG		0.1553926	1.1681165
22	U -Alb		-0.1767527	0.8379870
23	U -Krea	U -Cr	0.5905415	1.8049655
24	Hepat-B		-0.3764771	0.6862748

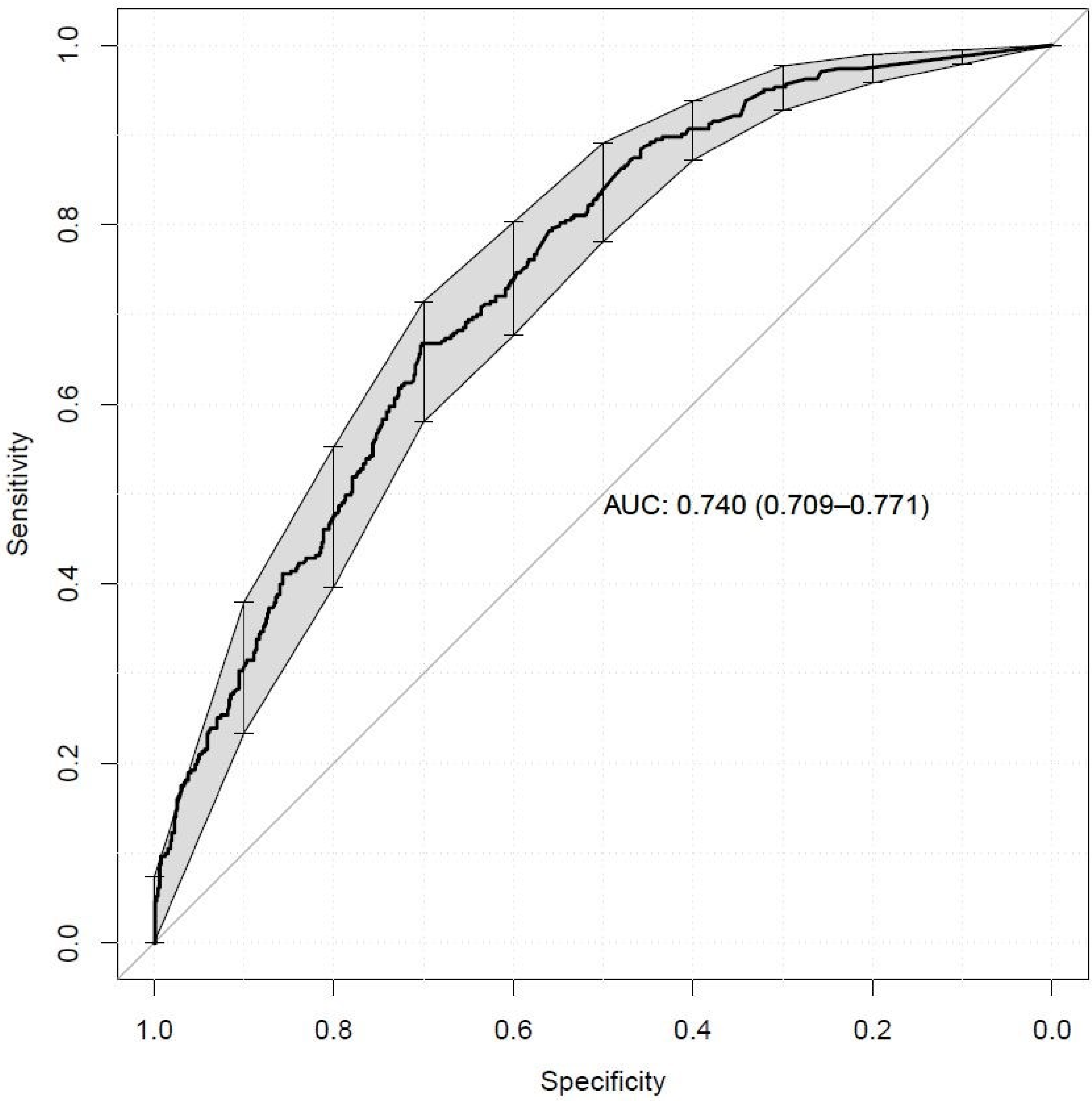

ROC model 1.

2. Frequency

Variable no.	Variable	Engl. Abbr.	Coefficient	OR
1	B -Eryt	B -RBC	0.0000595	1.0000596
2	B -HKR	B -HCT	0.0010154	1.0010159
3	B -Leuk	B -WBC	0.0006160	1.0006162
4	E -RDW		0.0002844	1.0002844
5	fP-Gluk	fP-Glu	0.0118636	1.0119343
6	P -CK		0.0202046	1.0204101
7	P -CK-MBm		0.0575010	1.0591863
8	P -IgG		-0.0006220	0.9993782
9	P -Myogl		0.0176269	1.0177831
10	P -Uraat	P -URA	-0.0048267	0.9951850
11	S -CDT		0.1529541	1.1652715
12	Hepat-A		0.3699369	1.4476432
13	S -Korsol	S -Cort	-0.0282894	0.9721070
14	U -Alb		-0.0211309	0.9790908
15	U -Krea	U -Cr	0.0008252	1.0008256
16	Hepat-B		-0.0189356	0.9812425

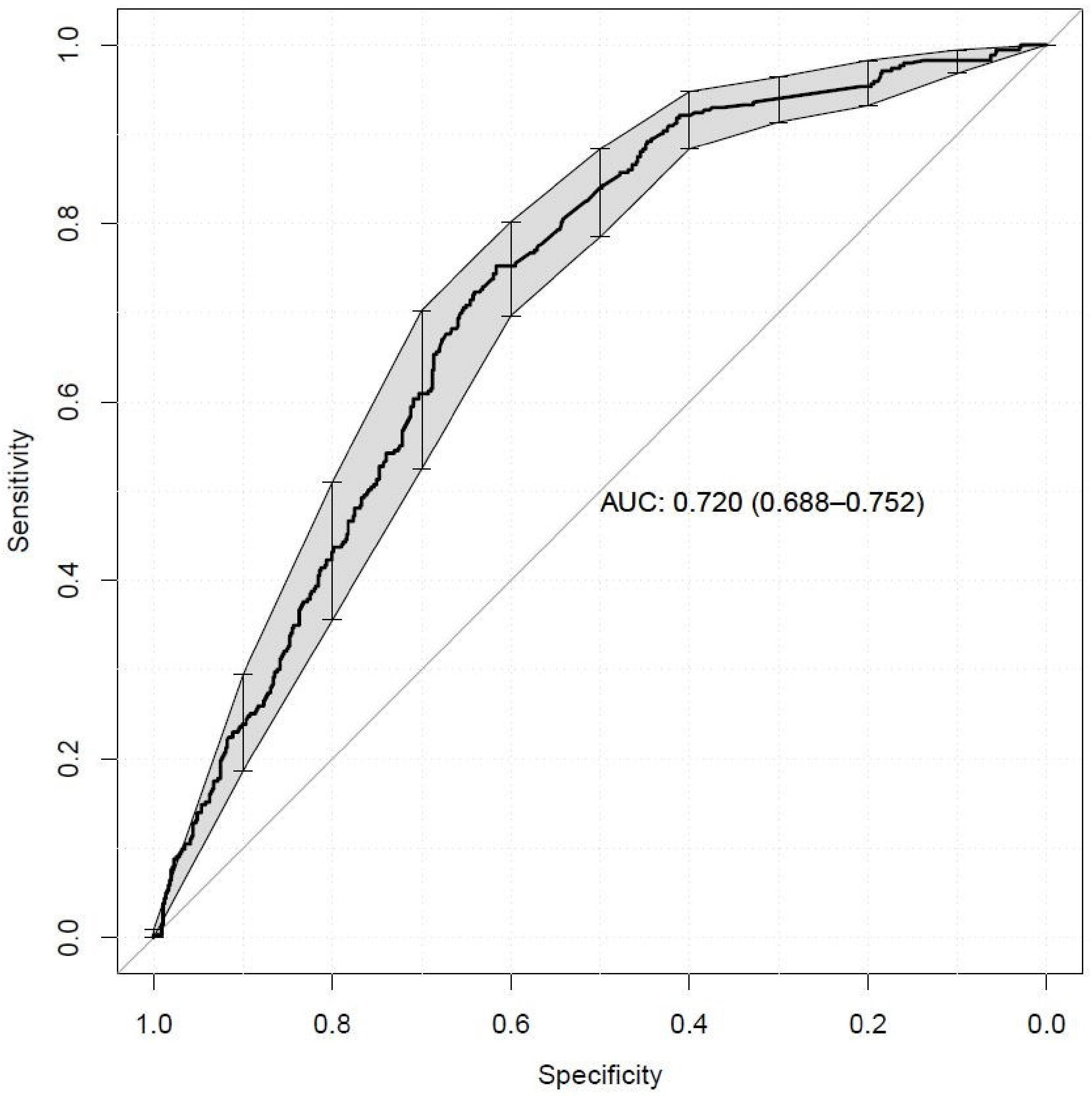

ROC model 2.

3. Variance

Variable no.	Variable	Engl. Abbr.	Coefficient	OR
1	B..Eryt(0.112, Inf]	B -RBC	0.2332762	1.2627301
2	B..HKRNA	B -HCT	-0.1223659	0.8848245
3	E..RDW(0.27, Inf]		0.6932761	2.0002579
4	fP.PTHNA		0.3881751	1.4742879
5	P..APTT(7.93, Inf]		-0.0884163	0.9153797
6	P..CK(6.15e+04, Inf]		0.1995641	1.2208704
7	P..CKNA		-0.1251462	0.8823679
8	P..IgA(0.157, Inf]		-0.4288822	0.6512366
9	P..Krea(83.4, Inf]	P -Cr	0.5657113	1.7606998
10	P..LDNA		0.1384546	1.1484976
11	P..NaNA		-0.1946363	0.8231340
12	P..UraatNA	P -URA	0.1773771	1.1940814
13	S..KorsolNA	S -Cort	0.5281788	1.6958410
14	S..Testo_comb(43.5, Inf]	S -T*	0.1115160	1.1179716
15	U..AlbNA		0.5433858	1.7218267
16	U..KreaNA	U -Cr	-0.4204700	0.6567381

* S -Testo_comb = Serum testosterone (S -Testo) and Serum testosterone, by mass spectrometry (S -TestoMS) results combined

NA following variable name equals non-available variation.

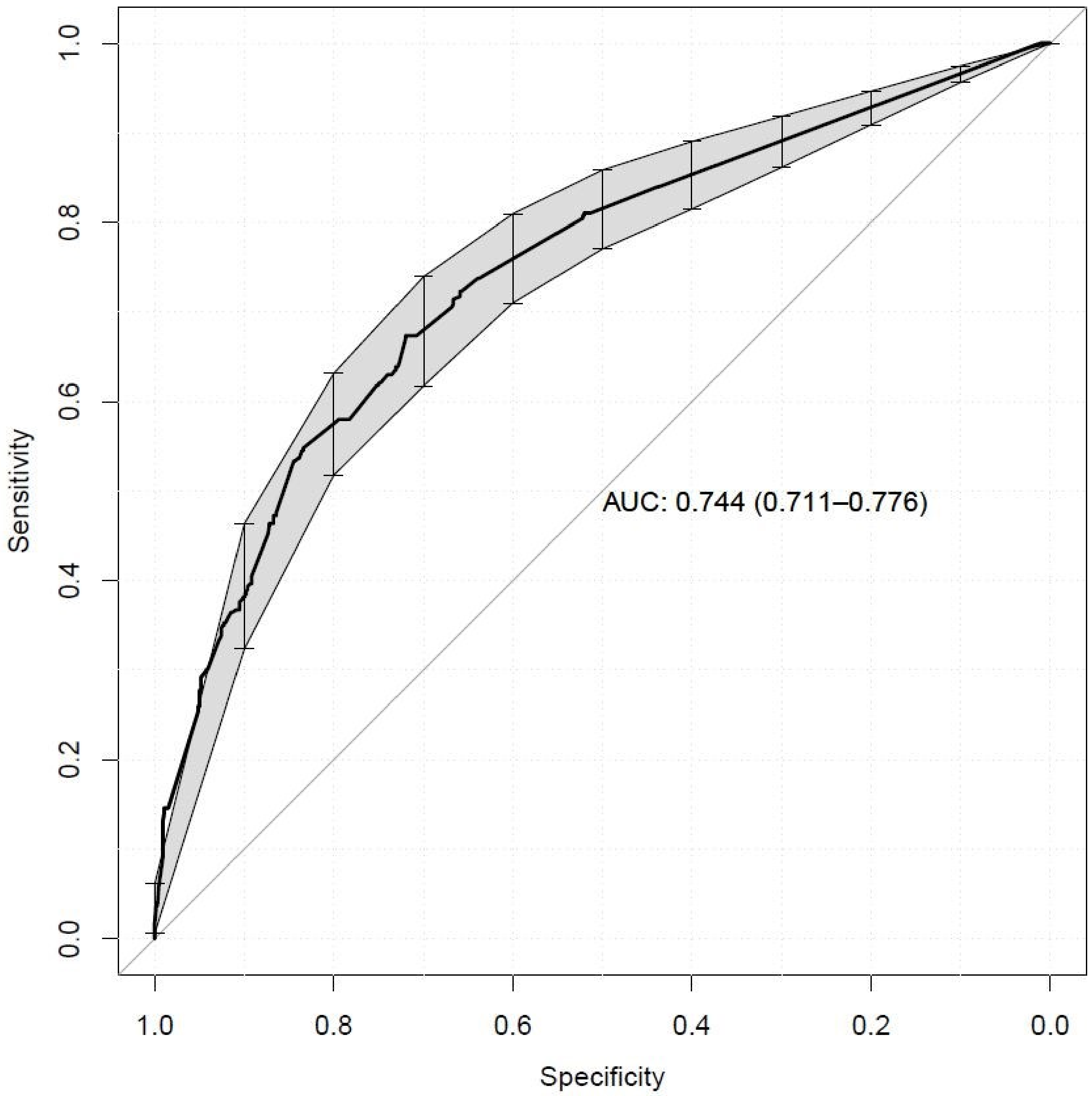
Sensitivity
1.0
0.8
0.6
0.4
0.2
0.0
Specificity
1.0
0.8
0.6
0.4
0.2
0.0
AUC: 0.744 (0.711–0.776)

ROC model 3.

4. Variance excluding NA

Variable no.	Variable	Engl. Abbr.	Coefficient	OR
1	B..Eryt(0.112, Inf]	B -RBC	0.2693702	1.3091397
2	B..Leuk(4.21, Inf]	B -WBC	0.0318196	1.0323312
3	E..RDW(0.27, Inf]		0.7163460	2.0469400
4	P..APTT(7.93, Inf]		-0.0750309	0.9277148
5	P..CK(6.15e+04, Inf]		0.3422836	1.4081597
6	P..IgA(0.157, Inf]		-0.5734366	0.5635853
7	P..Krea(83.4, Inf]	P -Cr	0.5917171	1.8070886
8	S..Korsol(2.27e+04, Inf]	S -Cort	-0.0021659	0.9978365
9	U..Alb(6.46e+03, Inf]		-0.3063478	0.7361305
10	U..Krea(23.8, Inf]	U -Cr	0.3051951	1.3568897

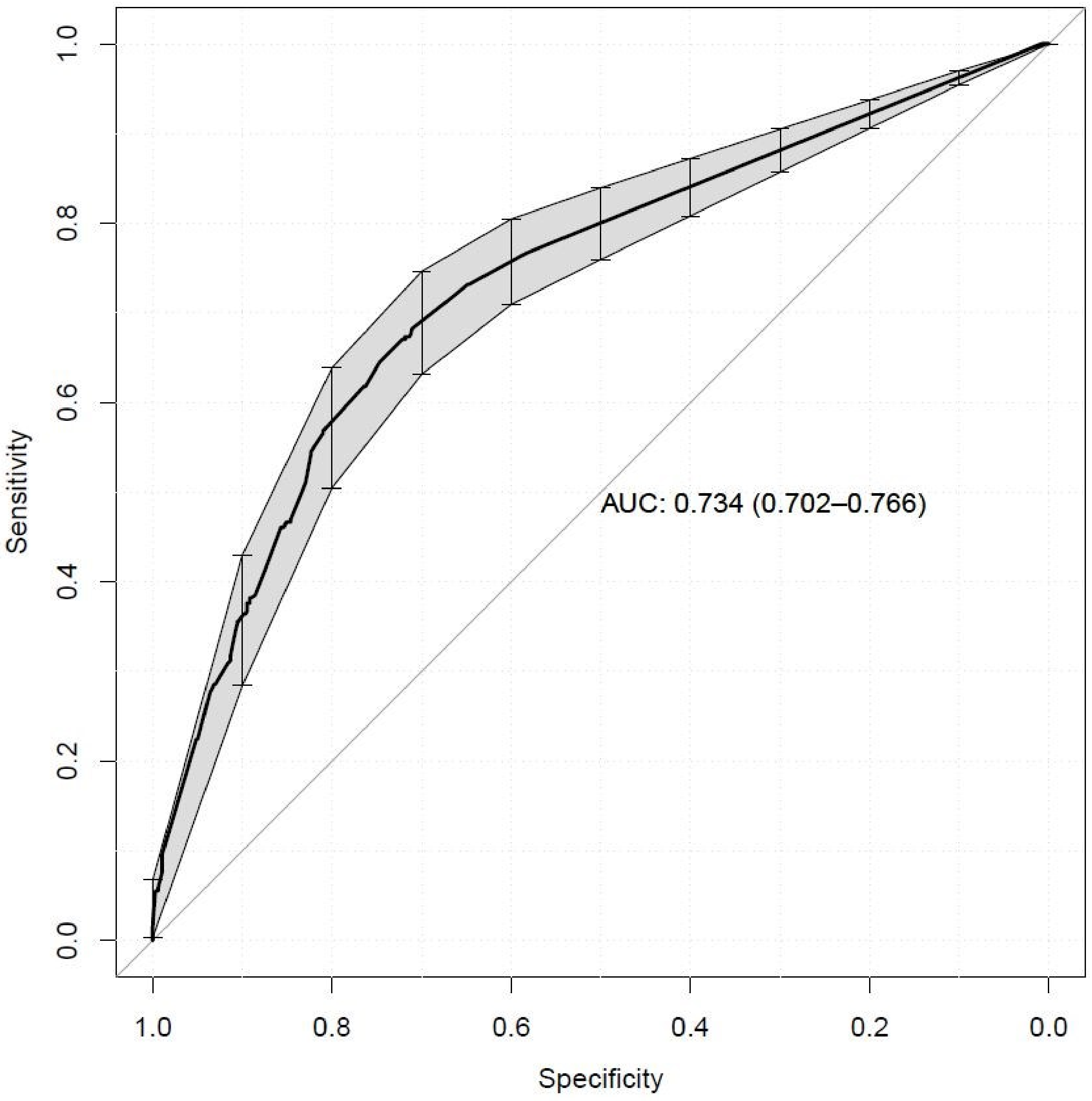

ROC model 4.

5. Indicator out of reference range

Variable no.	Variable	Engl. Abbr.	Coefficient	OR
1	B -Hb	B -HGB	0.3405275	1.4056889
2	B -HKR	B -HCT	0.2350016	1.2649108
3	E -MCH		0.0449683	1.0459947
4	E -RDW		0.9955003	2.7060780
5	fP-Fe		-0.1179671	0.8887253
6	fP-Kol	fP-TC	-0.0236058	0.9766706
7	fP-Kol-HDL	fP -HDL-C	0.1805833	1.1979159
8	fP-PTH		-0.6458213	0.5242318
9	fP-Trigly	fP -TG	-0.0766564	0.9262080
10	fS-Folaat	fS -Fol	-1.6265161	0.1966134
11	L -Baso(A)		-0.0112399	0.9888230
12	L -Mono(A)		-0.1775274	0.8373380
13	P -Amyl		0.0397780	1.0405797
14	P -CK		0.2276278	1.2556179
15	P -Ferrit		-0.0596256	0.9421172
16	P -FVIII.		-0.1039098	0.9013066
17	P -IgA		-0.9828164	0.3742556
18	P -IgM		-0.4848589	0.6157841
19	P -Krea	P -Cr	0.5165055	1.6761601
20	P -Myogl		0.0815447	1.0849617
21	P -Na		0.0072437	1.0072700
22	Pt-GFReEPI		0.0498477	1.0511110
23	S -CDT		0.1739019	1.1899388
24	Hepat-A		0.5260544	1.6922423
25	S -Korsol	S -Cort	-0.9052095	0.4044572
26	S -LH		0.2460230	1.2789290
27	S -Prot		-0.1302692	0.8778591
28	S -Testo_comb	S -T*	0.1558755	1.1686807
29	U -AlbKre	U -Acr	-0.2357060	0.7900129
30	U -Krea	U -Cr	0.4155492	1.5152027
31	Hepat-C		0.5262277	1.6925356

* S -Testo_comb = Serum testosterone (S -Testo) and Serum testosterone, by mass spectrometry (S -TestoMS) results combined

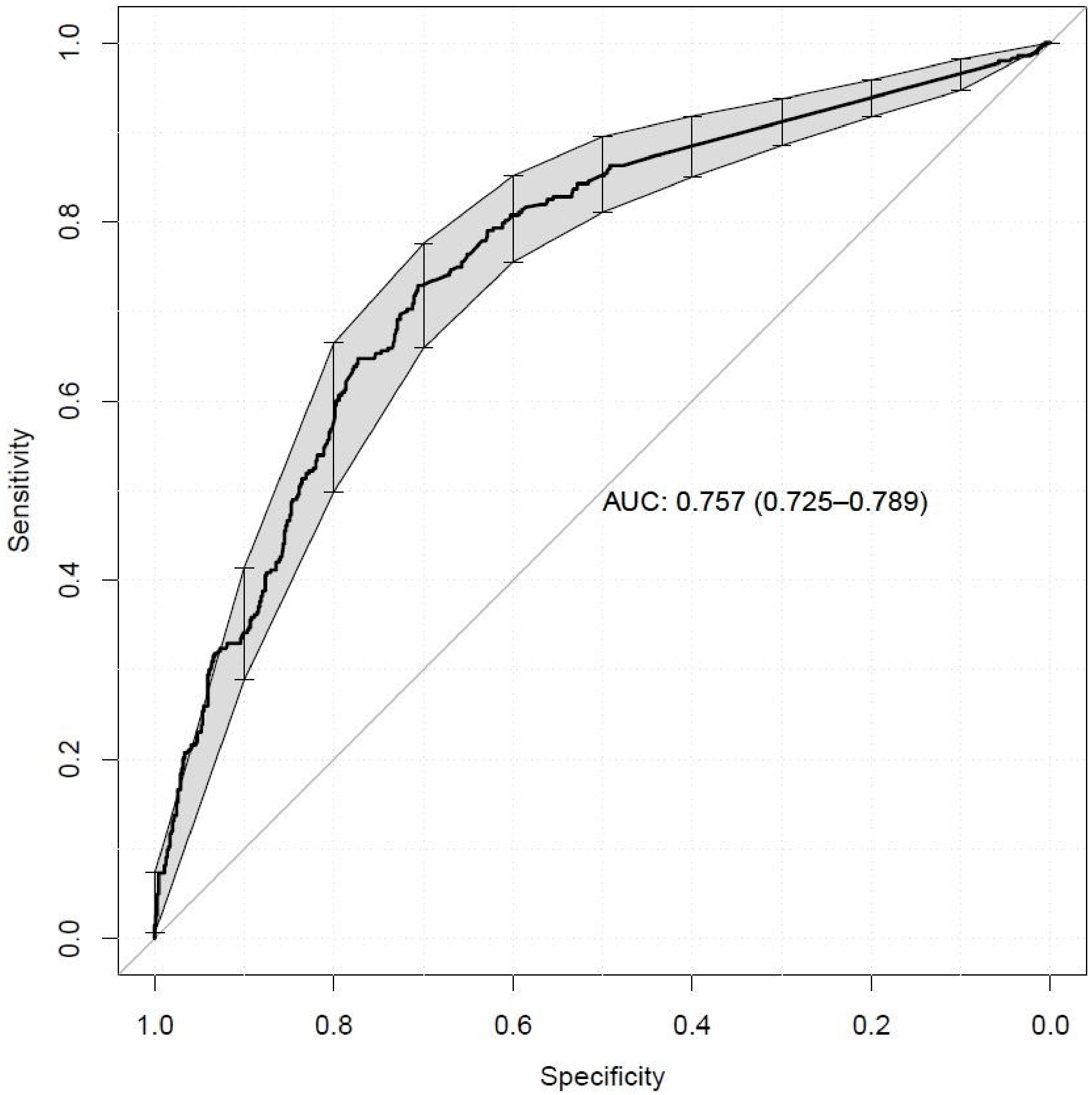

ROC model 5.

6. Frequency out of reference range

Variable no.	Variable	Engl. Abbr.	Coefficient	OR
1	B -HbA1c_comb	B -HbA1c*	-0.0033732	0.9966325
2	B -Leuk	B -WBC	0.0042145	1.0042234
3	fP-Kol-HDL	fP -HDL-C	0.0342961	1.0348909
4	P -ASAT	P -AST	0.0057346	1.0057511
5	P -CK		0.0136515	1.0137451
6	P -CK-MBm		0.0222882	1.0225384
7	P -IgA		-0.0454123	0.9556034
8	P -Myogl		0.0418837	1.0427732
9	P -Na		0.0077378	1.0077678
10	S -Korsol	S -Cort	-0.0556326	0.9458865
11	S -LH		0.1232800	1.1312012
12	U -AlbKre	U -Acr	-0.0121017	0.9879712
13	U -Krea	U -Cr	0.1111886	1.1176057
14	Hepat-C		0.0368500	1.0375374

*B -HbA1c_comb = Blood glycosylated hemoglobin (B -GHbA1CM, B -HbA1c) and Blood glycosylated hemoglobin, finger prick test (B -HbA1cVT) results combined; using the formula B -HbA1c (mmol/mol) = B -GHbA1CM(%) x 10.93 - 23.50

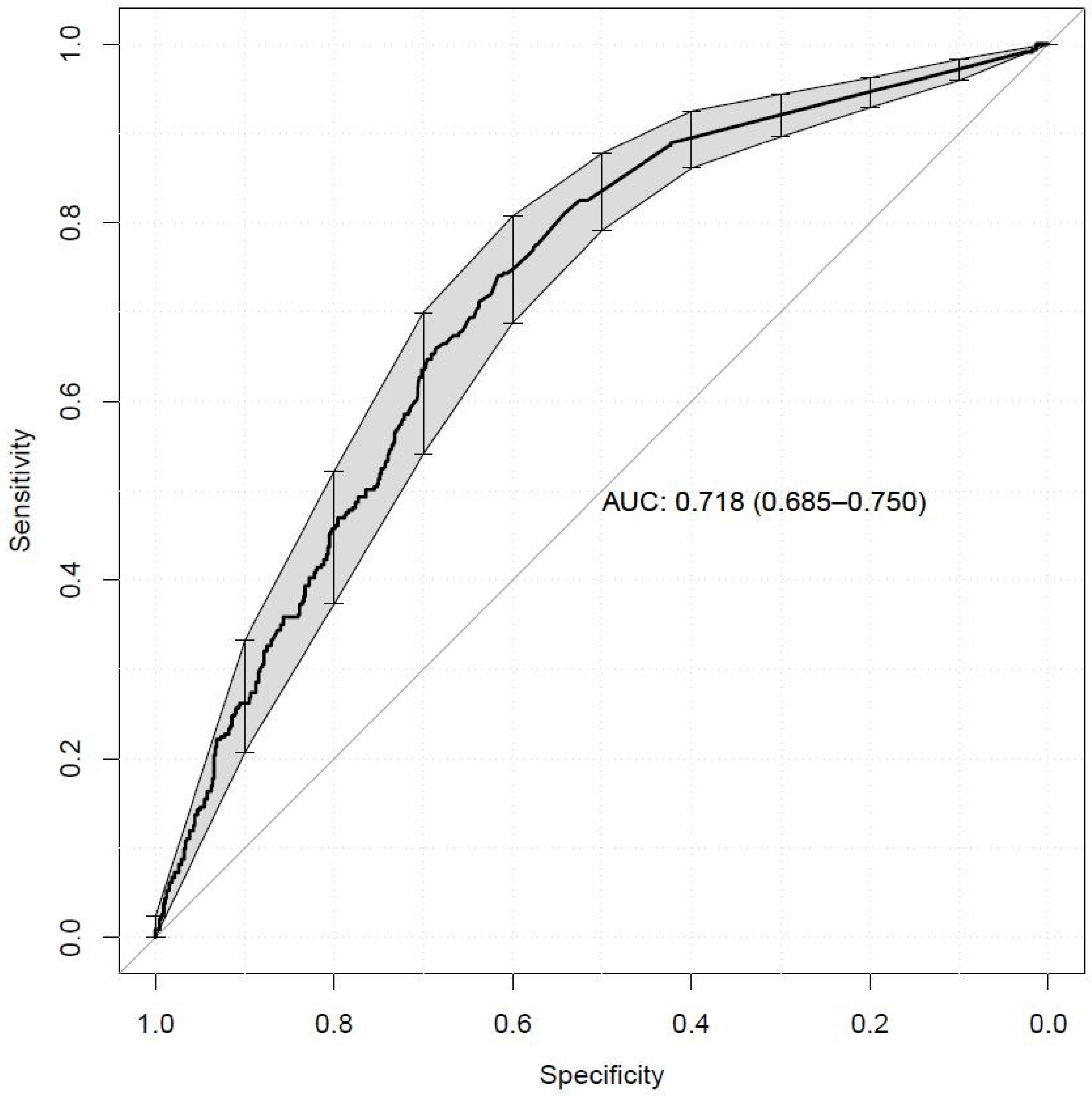
1.0
0.8
0.6
0.4
0.2
0.0
Sensitivity
AUC: 0.718 (0.685–0.750)
1.0
0.8
0.6
0.4
0.2
0.0
Specificity

ROC model 6.
